# Seasonal and spatial dynamics of the planktonic trophic biomarkers in the Strait of Georgia (northeast Pacific) and implications for fish

**DOI:** 10.1038/s41598-020-65557-1

**Published:** 2020-05-22

**Authors:** David Costalago, Ian Forster, Nina Nemcek, Chrys Neville, R. Ian Perry, Kelly Young, Brian P. V. Hunt

**Affiliations:** 1 Institute for the Oceans and Fisheries, University of British Columbia, AERL, 2202 Main Mall, Vancouver, BC V6T 1Z4 Canada; 2 Pacific Science Enterprise Center, Fisheries and Oceans Canada, West Vancouver, BC V7V 1N6 Canada; 3 Institute of Ocean Sciences, Fisheries and Oceans Canada, Sidney, BC V8L 4B2 Canada; 4 Pacific Biological Station, Fisheries and Oceans Canada, Nanaimo, BC V9T 6N7 Canada; 5 Department of Earth, Ocean and Atmospheric Sciences, University of British Columbia, 2020 – 2207 Main Mall, Vancouver, BC V6T 1Z4 Canada; 6 Hakai Institute, Tula Foundation, PO Box 309, Heriot bay, BC V0P 1H0 Canada

**Keywords:** Biogeochemistry, Biooceanography, Conservation biology, Ecophysiology, Ecosystem ecology, Stable isotope analysis, Marine biology

## Abstract

Fish growth and survival are largely determined by the nutritional quality of their food, and the fish that grow quickly during early life stages are more likely to reproduce. To adequately estimate the quality of the prey for fish, it is necessary to understand the trophic links at the base of the food-web. Trophic biomarkers (e.g., stable isotopes and fatty acids) are particularly useful to discriminate and quantify food-web relationships. We explored the connections between plankton food-web components, and the seasonal and spatial dynamics of the trophic biomarkers and how this determines the availability of high-quality prey for juvenile Pacific salmon and Pacific herring in the Strait of Georgia, Canada. We demonstrate that the plankton food-web in the region is largely supported by diatom and flagellate production. We also show that spatial differences in terms of energy transfer efficiency exist in the region. Further, we found that the fatty acid composition of the zooplankton varied seasonally, matching a shift from diatom dominated production in the spring to flagellate dominated production in the summer. This seasonal shift conferred a higher nutritional value to zooplankton in the summer, indicating better quality prey for juvenile salmon and herring during this period.

## Introduction

The growth and survival of fish are conditioned by the amount and nutritional content of their food. Fish that grow quickly during critical growth periods have higher survival rates than fish with slower growth because they can better escape predators, outcompete competitors, or endure adverse conditions^1,2^. Since zooplankton are the principal dietary group for juvenile salmon and forage fish such as herring, variations in the temporal and spatial dynamics of the nutritional composition and abundance of zooplankton are therefore critical factors for their growth and survival^3^.

The trophic structure of planktonic food webs is expected to play a key role in their nutritional dynamics as well as energy transfer efficiency^4^. Environmental conditions, such as nutrient supply, underpin these pathways and provide a mechanistic linkage between ocean conditions and the nutritional health of zooplanktivorous fish^5^. Identifying trophic interactions is therefore an important step towards understanding drivers of zooplanktivorous fish survival.

Trophic biomarkers such as fatty acids (FAs) and stable isotopes are particularly useful to discriminate and quantify trophic relationships. Marine primary producers have unique FA patterns that may be transferred conservatively to primary consumers (see examples and references in Table 1). Consequently, FAs are often used as dietary tracers in the food-web while also being indicators of food quality^6,7^.

**Table 1:** Fatty acid markers selected for this study.

Fatty acid / trophic marker	Source/index	Reference
16:0	Long-term energy storage, herbivory	Daalsgard *et al*. (2003)
18:1n7	Bacteria	Stevens *et al*. (2004)
18:1n9	Trophic level, carnivory	Daalsgard *et al*. (2003), Stevens *et al*. (2004)
18:2n6	Terrestrial detritus	Daalsgard *et al*. (2003), Henderson & Tocher (1987)
18:3n3	Green algae	Li *et al*. (2002)
20:5n3	Diatoms	Mayzaud *et al*. (1990)
22:6n3	Flagellates	Daalsgard *et al*. (2003)
22:6n3/20:5n3 (DHA/EPA)	Food quality, flagellates	Sargent and Lee (1975), Parrish *et al*. (2015), Budge and Parrish (1998)

The nutritional quality of zooplankton prey is often defined by their biochemical composition with respect to essential FAs that are required by fish for growth and physiological performance. Essential FAs are only synthesized by primary producers, and fish are dependent on the transfer of the FAs through the food web in order to meet their metabolic requirements^8^. In particular, n3 and n6 type polyunsaturated FAs (PUFAs) play a role in all animals’ health^9^, and the concentration of dietary essential FAs is correlated with the somatic growth of zooplankton^10^. The PUFAs 20:5n3 (eicosapentanoic acid, EPA) and 22:6n3 (docosahexanoic acid, DHA) are known to be particularly important for marine fish, since these FAs are essential for cell development and brain function^11^. However, the ability of fish to biosynthesize EPA and DHA is limited. Thus, fish must acquire these FAs through their diet, and the ratio DHA/EPA is often used as a proxy for the nutritional quality of plankton^12,13^. Although individual FAs are good indicators of food quality, these compounds become increasingly modified with each step in the food chain and are therefore not ideal on their own to define linkages between trophic levels^11^.

The stable isotope ratios of carbon (δ^13^C) and nitrogen (δ^15^N) in an organism’s tissues provide an additional means to identify an organism’s dietary sources and food-web position, respectively^14^. In particular, δ^15^N indicates trophic position, as isotopic fractionation leads to an increase of the N isotope values with increasing trophic levels^15^. δ^13^C, on the other hand, exhibits lower fractionation and hence it can be used to determine carbon sources in food-webs and to discriminate between photosynthetic pathways (e.g., C3 versus C4 photosynthetic pathways in plants, and freshwater versus marine primary producers)^16^. In combination, FA and stable isotope analyses represent a powerful tool for determining complex food-web linkages and the varying contribution of primary producers^7,17,18^.

The Strait of Georgia (SoG) is a large estuarine system in British Columbia, Canada, which receives significant inputs of freshwater from the Fraser River and other smaller rivers^19^. The SoG represents a critical habitat on the migration route for some of Canada’s most important Pacific salmon stocks^20–22^. Most juvenile salmon enter the SoG in March-July, and spend variable amounts of time in the strait before moving to the outer coast and open ocean^22,23^. The SoG is also the spawning and rearing ground for British Columbia’s largest herring stock^24^. Pacific herring are an important species in the northeast Pacific because of their abundance, their high nutritional value, and their central position in the pelagic food-web - herring play an essential role in transferring energy from planktonic producers to top predators^25^. Moreover, Pacific salmon and herring are both key commercial, recreational, and aboriginal fish stocks in the SoG^22–24^. Environmental conditions in the SoG have undergone significant changes over the past few decades, including a warming trend^26^, increasing variability of the spring phytoplankton bloom timing^27^, and shifts in zooplankton community composition^28,29^. Long-term changes in oceanographic conditions and plankton are considered to have been a key factor in the fluctuating productivity of Pacific salmon species (i.e., chinook salmon *Oncorhynchus tshawytscha*, chum salmon *O. keta*, coho salmon *O. kisutch*, pink salmon *O. gorbuscha*, and sockeye salmon *O. nerka*) and Pacific herring (*Clupea pallasi*) in the northeast Pacific Ocean^30^. More recently, the North Pacific warm water anomaly known as the ‘Blob’ resulted in extreme temperature events in the northeastern Pacific during 2014–2015^31^ and had many ecosystem consequences including being the likely driver of the early spring bloom in the SoG in 2015^32^.

How plankton food-webs respond to changing environmental conditions is largely mediated by the complex interactions between physical conditions, chemical components and the different plankton groups^33^. This study combines the analysis of size-structured plankton food-webs with a suite of biochemical methods (fatty acids and stable isotopes) in order to identify food sources and trophic links to juvenile salmon and herring in the SoG. Specifically, we analyse the spatial and temporal dynamics of trophic biomarkers in the plankton food web and its effect on the nutritional quality of zooplankton in the prey field of zooplanktivorous fish. Finally, we discuss the implications of these dynamics for juvenile Pacific salmon and herring health and productivity.

## Results

### Fatty acid composition of particulate organic matter and zooplankton size classes

Although the nMDS ordination of zooplankton FA composition (in percentage) showed a weak separation between different regions, seasons and size classes (Suppl. Fig. 1), a more detailed analysis revealed some statistical differences among groups. In particular, the perMANOVA test showed that the FA composition of particulate organic matter (POM) and zooplankton differed significantly (p < 0.001) among all tested factors (i.e., region, season and size) (Table 2). In particular, the perMANOVA output revealed that ${F}_{region}$ and ${r}_{region}^{2}$ were relatively small compared to *F* and *r*^2^ for the season and size groups (Table 2). The FA composition of the samples also differed significantly for the interactions region–season, region-size and season-size (Table 2). The Tukey HSD tests showed that FA compositions of POM and zooplankton were significantly different between summer and the other seasons (p < 0.001), whereas no significant differences were detected between individual regions or between zooplankton size classes (Table 3).

**Table 2:** Results from 3- factor permutational analysis of variance model (perMANOVA) testing the effect of region, season and size class (POM, and small, medium and large zooplankton) on the overall FA composition of POM and zooplankton.

	df	SS	MS	F Model	R^2^	*p*-value
season	2	0.5787	0.28935	15.421	0.03094	0.0001
region	2	0.5097	0.25483	13.581	0.02725	0.0001
size	3	2.0069	0.66896	35.652	0.10731	0.0001
season:region	4	0.3276	0.08191	4.365	0.01752	0.0001
season:size	5	0.5418	0.10837	5.775	0.02897	0.0001
region:size	6	0.2498	0.04163	2.219	0.01336	0.0013
season:region:size	10	0.1701	0.01701	0.907	0.0091	0.6616
Residuals	763	14.3167	0.01876		0.76554	

Abbreviations: df = degrees of freedom, SS = sums of squares, MS = mean square error.

**Table 3:** Results of 2-way ANOVAs and tests of the differences in DHA/EPA of POM and of each zooplankton size class between seasons, regions and the interaction between both factors.

		POM	Small	Medium	Large
region	F	3.23	3.24	14.97	4.06
	*p*	0.08	**0.04**	**<0.001**	**0.02**
			South-Central	North-Central, North-South	North-Central
season	F	2.63	10.91	34.25	65.96
	*p*	0.13	**<0.001**	**<0.001**	**<0.001**
			Summer-Spring	Spring-Winter, Spring-Summer	Spring-Winter, Spring-Summer
region:season	F	0.14	1.3	1.99	3.38
	*p*	0.87	0.27	0.09*	**0.01**
				Central: Spring-Winter, Spring-Summer; North: Spring-Winter; South: Spring-Summer; Summer: North-Central	Differences between spring and summer were significant in all regions

Significant differences between particular seasons, regions and their interactions were tested with Tukey HSD post hoc tests, and only combinations where *p* < 0.05 are presented in the table (*although the overall interaction region:season was not significant for the medium size zooplankton (*p* = 0.09), the Tukey HSD test output did show significant differences for the one-to-one interactions within each particular region season as detailed below).

The SIMPER analysis showed that the FAs 18:1n9, 22:6n3 (DHA), 20:5n3 (EPA), 14:0 and 16:0 contributed the most to differences between regions and plankton size groups (Suppl. Table S2). The FAs contributing the most to the differences in FA composition between seasons were 18:1n9, DHA, EPA, 14:0, 16:0 and 20:1n9 (Suppl. Table S2). Therefore, we explored how the concentration and the percentage of 16:0, 18:1n9, 18:1n7, 18:2n6, 18:3n3, EPA and DHA, as well as the ratio of DHA to EPA (DHA/EPA), correlated with the region, plankton size and season groups, and in the species-specific samples (Table 1).

The total FA concentration in the POM samples ranged from 0.03 to 0.45 mg/g and increased from south to north (Suppl. Fig. S2). FA concentration in zooplankton samples ranged from 0.01 to 187.6 mg/g. The mean values in the small, medium and large size fractions were 16.12, 21.72 and 13.25 mg/g, respectively (Suppl. Table S3), and the value for medium size zooplankton was significantly (*p* < 0.001) larger than for the other two size fractions. The total FA concentration of small and medium sized zooplankton showed a non-significant decrease from south to north, while large zooplankton showed a significant (*p* = 0.02) increase with latitude (Suppl. Fig. S2). All zooplankton size groups had approximately ten times lower FA concentration in winter (0.03 and 8 mg/g) than in spring (0.01 and 99.2 mg/g) and summer (0.12 and 98 mg/g) (Fig. 2, Suppl. Table S3).

The percentage of 16:0 was not significantly different between seasons but was significantly higher in POM (~31% average of all seasons) than in zooplankton. The percentage of 16:0 was significantly higher in the large zooplankton fraction (~20.5%) than in the small and medium size fractions (~18% and ~17%, respectively) (Fig. 2). Similarly, 18:1n7 (bacteria tracer) and 18:1n9 (carnivory tracer) percentages did not show significant seasonal differences. The percentage of 18:1n7 was significantly higher in large zooplankton in summer and in spring (4.82% and 4.26%, respectively) than in small (3.21% and 2.52%) and medium size zooplankton (3.08% and 2.82%) (Fig. 2). The percentage of 18:1n9 was always significantly higher in large zooplankton than in the other zooplankton size fractions and POM, reflecting higher carnivory in this group (Fig. 2). Percentage of 18:2n6 (terrestrial detritus) was significantly higher in zooplankton in spring (2.83% average of all sizes) than in winter (2.18%) and summer (2.63%), with large zooplankton having the lowest percentage (Fig. 2). The contribution of 18:2n6 was about two times higher in POM than in zooplankton and was highest in the summer. Of the selected FAs, 18:3n3 (green algae) had the lowest proportion in all size groups and seasons; this FA had higher levels in POM than in the zooplankton during the spring and, particularly, the summer seasons (Fig. 2). DHA (flagellates) levels were significantly higher in summer (14.49% on average across all sizes) than in spring (11.81%) and winter (13.92%), with % values in POM being generally two to three times lower than in zooplankton (Fig. 2). EPA (diatoms) had higher concentrations in spring (16.67% on average across all sizes) than in summer (14.93%) and winter (14.70%), with % values in POM being generally two to three times lower than in zooplankton (Fig. 2). Consequently, DHA/EPA values were significantly lower in spring (<1 for POM and all zooplankton sizes) than in summer and winter (Fig. 2).

DHA/EPA in POM was not significantly different between seasons nor between regions. We found statistically significant differences in DHA/EPA of small zooplankton between seasons (higher in summer than in spring) and between regions (South being the lowest) but not when looking at the interactions between these two factors (Table 3; Suppl. Table S3). DHA/EPA in medium size zooplankton was significantly lower in the North region compared to the other two regions, and it was also significantly lower in the spring compared to summer and winter (Table 3; Suppl. Table S3). In the large zooplankton size fraction, DHA/EPA was significantly higher in the Central region than in the South and North regions, and it was up to almost 100% higher in the summer than in the spring in all regions (Table 3; Suppl. Table S3).

### Zooplankton species-specific fatty acid composition

The copepod species identified (i.e., *Calanus* spp. - a mix of *C. marshallae* and *C. pacificus* -, *Eucalanus bungii*, *Neocalanus* sp. and *Paraeuchaeta elongata*) and the amphipod *Cyphocaris challengeri* had the highest FA concentration of all the analyzed taxa (Fig. 3), with average values>15 mg/g. Gelatinous zooplankton (i.e., the groups ‘jelly mix’, *Beroe* sp. and *Pleurobrachia bachei*) had the lowest FA concentration of all groups, with values <1 mg/g (Fig. 3). *E. bungii* had the highest percentage of 16:0 (28.8%), whereas another copepod, *P. elongata*, had the lowest percentage (6.8%) (Suppl. Fig. S3). Decapods and euphausiids had the highest percentage of 18:1n7 (>5%) of all taxa, and *Beroe* sp., *P. bachei* and the copepods *P. elongata* and *Calanus* spp. had the lowest percentages (<1.6%) of this FA (Suppl. Fig. S3). The highest percentage of 18:1n9 was found in *P. elongata* (34%) and *C. challengeri*, (27.7%) whereas the lowest values corresponded to *Clione limacina* (2.97%), *P. bachei* (4.16%) and *Calanus* spp. (4.2%) (Suppl. Fig. S3). Although large differences were not detected in the percentage of 18:2n6 between species (Suppl. Fig. S3), *Euphausia pacifica* as well as copepods such as *Calanus* spp. and *E. bungii* had the highest values for this FA. The percentage of 18:3n3 was highest in the molluscs (i.e., *C. limacina*, *Limacina helicina* and Octopoda), and lowest in the copepod groups (e.g., *E. bungii*, *Calanus* spp. and *Neocalanus* sp.) (Suppl. Fig. S3).

The percentage of EPA was particularly high (>20%) in decapods, euphausiids and *Beroe* sp., and it was lower (<15%) in fish larvae, *P. elongata*, *P. bachei*, Conchoecinae and *C. challengeri*. Octopoda, fish larvae and chaetognaths were the taxonomic groups with a higher DHA percentage (25.4%, 24.8% and 23.6%, respectively), whereas the copepods *Calanus* spp. and *E. bungii* had the lowest levels of DHA (4.6% and 3.15%, respectively) (Suppl. Fig. S3). Fish larvae were the group with the highest mean DHA/EPA value (1.85), followed by the copepod *P. elongata* (1.48), Octopoda (1.42), the polychaete *Tomopteris* spp. (1.4) and chaetognaths (1.39) (Fig. 3). The lowest DHA/EPA values were measured in the copepods *E. bungii* and *Calanus* spp. (0.16 and 0.28, respectively) (Fig. 3).

DHA and EPA were the most abundant FAs of the five selected trophic markers in all the species analysed (Fig. 4). In general, the euphausiid *E. pacifica*, copepods *Neocalanus* spp. and *E. bungii*, and the amphipod *C. challengeri* were among the species with the lower percentage of the five selected markers combined in spring and in summer. Octopoda, chaetognaths and fish larvae contained a higher percentage of DHA and of all five selected FAs combined than most other taxonomic groups in both seasons (Fig. 4).

### Carbon and nitrogen isotopic composition of POM and zooplankton

The δ^13^C value of POM and small and large zooplankton decreased with latitude (Suppl. Fig. S4), although the trend was only significant (p = 0.01) for small zooplankton. Medium size zooplankton δ^13^C showed a slightly increasing trend with latitude. The δ^15^N value had a significant (p < 0.05) positive correlation with latitude for POM and the three zooplankton size fractions (Suppl. Fig. S4).

In general, all size fractions of plankton had the lowest δ^13^C values in winter, whereas the highest δ^13^C values were in spring (Suppl. Table. S4). There were no evident seasonal differences in the δ^15^N signatures and trophic level of the plankton size fractions (Suppl. Table. S4). The δ^13^C values of POM were significantly different (p < 0.05) between spring and summer only in the South region (Fig. 5). The δ^13^C values of small zooplankton were significantly different (p < 0.005) between the three seasons only in the Central region (Fig. 5). The δ^13^C values of medium size zooplankton showed significant seasonal differences in the North and Central regions; in particular, spring and winter were very different in the North (p < 0.001), whereas in the Central region there were statistical differences between all seasons, with the lowest δ^13^C in the winter and the highest δ^13^C in the summer. Large zooplankton δ^13^C values in the North and Central regions were significantly higher in spring compared to the other two seasons (Fig. 5).

The isotopic analyses of individual zooplankton species showed that the studied species had higher δ^13^C in spring than in summer, with no clear seasonal differences in δ^15^N (Suppl. Fig. S5). Most amphipod species had the highest δ^13^C of all analyzed taxa (i.e., *Themisto pacifica*, *C. challengeri* and *Scina borealis*, with values of -16.9‰, -17.4‰ and -17.5‰, respectively), while the lowest C isotopic values were found in *E. pacifica* (-20.28‰) (Suppl. Fig. S5). In terms of δ^15^N, the highest values were observed in chaetognaths (12.95‰), fish larvae (12.69‰) and amphipod species (>10‰), while decapod zoea had the lowest values (4.94‰) (Suppl. Fig. S5).

## Discussion

In this study we examined the trophic pathways in a productive temperate ecosystem, using biochemical tracers (i.e., FAs and bulk C and N isotopes) to resolve linkages between POM and zooplankton size fractions and species. We found that seasonality was the dominant driver of variability in plankton FA composition, and largely reflected the seasonal succession of the phytoplankton. Analysis of the phytoplankton community composition from 2018 in the SoG showed that diatoms dominated in spring but were almost entirely substituted by flagellates in the summer^34^. It is generally assumed that DHA/EPA > 1 indicates a dominance in the contribution of flagellates, whereas a value <1 suggests a greater contribution of diatoms^35^. We showed that FAs in POM were largely dominated by the diatom FA marker EPA (i.e. DHA/EPA < 1) during spring. In the summer, following an intensification of stratification, the phytoplankton community became dominated by mixed flagellates (i.e. haptophytes, prasinophytes, cryptophytes, raphidophytes and dinoflagellates)^34^. This was reflected by the increase in the proportion of DHA in POM and all zooplankton size-fractions in the SoG.

EPA may be of general importance for trophic transfer efficiency in aquatic food-webs^10^. This FA is highly retained in zooplankton, especially in larger zooplankters, whereas DHA is highly retained in fish^8^. DHA is particularly important in the early development of fish^36,37^, and juvenile salmon tend to retain DHA much more than other FAs^8,38,39^. However, the most DHA-rich zooplankton groups of this study (i.e., fish larvae, *P. bachei*, *P. elongata*, Octopoda, *Tomopteris* spp., chaetognaths) are not reported to be common prey for juvenile salmon (except in the case of herring larvae as prey for chinook salmon in spring^40^) and herring in the SoG. Some of these zooplankton groups might not be available to herring and salmon because of their different vertical distributions in the water column or because of their seasonal dynamics; those with soft bodies may also be quickly digested and expelled from fish stomachs. For example, once in the sea most juvenile salmon are found in the upper 10–20 m of the water column^41^ and herring in the SoG tend to occupy coastal, shallow waters^42^, whereas larger zooplankton (i.e., large copepods, chaetognaths, etc.) usually remain below 50 m depth during the day^29,33^.

When compared to other regions at similar latitudes, the copepod DHA/EPA levels in the SoG appear to be lower. For instance, Bevan (2015)^43^ reported DHA/EPA values between 0.5–1.5 for *Calanus* spp. in 2010–2011 from the west coast of Vancouver Island. In the North Sea, the calanoid copepods *Acartia clausi* and *Temora longirostris* presented DHA/EPA values between 1.19–1.99, depending on the season^44^. It might be argued that the relatively low DHA/EPA in copepods in the SoG is related to a low DHA/EPA in phytoplankton. However, it has been shown that even with POM DHA/EPA ≤ 1, copepod DHA/EPA consistently ranged from 0.4–1.5^45^. Thus, the FA composition at the base of the food chain in the SoG should be adequate to provide zooplankton with higher DHA/EPA than found in this study. The reason behind the seemingly low DHA/EPA in the zooplankton in the SoG compared to other regions with similar POM DHA/EPA deserves further investigation, but the relatively high contribution of diatoms in the local food-web is likely to be an important factor^46^.

Pacific salmon and Pacific herring are richer in DHA than most fish species^47^ and may have adapted to select DHA-rich prey to fulfill their requirements for this FA^48^. In general, dietary DHA/EPA ≥ 2 is considered optimal for these fish species^49^. Our data showed that, among all the zooplankton species analyzed, only fish larvae and *P. elongata* could meet the requirements for an optimal diet for juvenile salmon and herring in the SoG. El-Sabaawi *et al*.^17^ calculated the DHA/EPA of four calanoid copepod species collected during May 2004–2006 in the SoG. They found that, similar to our data, *Neocalanus plumchrus*, *E. bungii*, and *C marshallae* generally had DHA/EPA < 0.5, whereas DHA/EPA of *P. elongata* was in the range of 1–1.5. In addition, a similar nutritional value for *N. plumchrus* (i.e., DHA/EPA < 0.5) was found in the SoG in 1996–1997^50^. This might suggest that DHA/EPA of zooplankton in the region has not varied substantially in the last two decades and thus the nutritional value of copepod prey for juvenile salmon and herring in the SoG has been suboptimal for at least 20 years. Present data cannot tell whether there has been historically (i.e., prior to 1996) relatively low DHA/EPA values in the zooplankton in the SoG, but it could be hypothesized that if a disruption in the FA composition and transfer from POM to zooplankton started occurring only in the past 2–3 decades, the factor(s) provoking the disruption might also be related to the general decline in the Pacific salmon populations and their smolt survival in Canada since the 1980s^51^.

Our analysis provided insights into several other aspects of the zooplankton food web. Particulate organic matter, dominated by phytoplankton, generally had a much larger proportion of 16:0 than zooplankton, possibly because this FA does not accumulate in consumers as much as other more complex FAs^11^. Thus, 16:0 in consumers can also be used as a proxy for herbivory. The copepod *E. bungii* has a particularly high proportion of 16:0 (28.8% of total FAs) while it also has the lowest percentage of DHA (2.78% of total FAs) and high EPA. Concurrently, this species had one of the lowest δ^15^N values (8.55‰). These results suggest that this species had a diet dominated by phytoplankton, unlike many copepod species that are omnivorous, and may thus be an important, albeit of relatively poor nutritional quality, link between primary producers and the fish that consume these copepods^52^.

Terrestrial matter can be relatively abundant in coastal and estuarine ecosystems, especially on the coast of British Columbia, which is influenced by high rainfall and freshwater/materials flux from the North Pacific Temperate Coastal Rainforest^53,54^. The SoG is additionally influenced by the significant outflow of the Fraser River, which peaks between May-July^55^. The contribution of terrestrial material to the SoG food-web is therefore expected to be high and seasonal. FA tracers have been effectively used to detect the input of freshwater organic matter into coastal food-webs^7,56,57^. In this study, we used 18:2n6 as a proxy for freshwater/terrestrial contribution to the SoG pelagic food web. POM showed a peak in 18:2n6 in summer which corresponded with the peak of Fraser River discharge. However, there was not a significant seasonal difference in zooplankton 18:2n6, indicating that terrestrial material made a small contribution to zooplankton diet. Terrestrial material contributions can be further assessed using stable isotopes, with δ^13^C values being significantly lower in terrestrial than marine organic matter (−28 to −30‰ compared with -20 to −24‰)^58^. In our study, δ^13^C values had a strong marine signature supporting the assessment from FA data that terrestrial contribution to the marine system was small.

Although the Tukey test showed no significant differences in the overall FA composition of the phytoplankton and zooplankton communities between the three regions within the SoG, we detected spatial patterns in some of the selected trophic markers. For example, the southern region had relatively lower levels of both carnivory and green algae biomarkers (i.e., lower percentages of 18:1n9 and 18:3n3, respectively) in all zooplankton size classes (although the relationship was not significant for 18:1n9 in large zooplankton) than the other regions. In addition, the values of EPA, DHA and DHA/EPA in the South were generally higher than in the Central and North regions (see Suppl. Fig. S2), supporting higher quality food for zooplanktivorous fish like salmon and herring in the southern part of the SoG.

A higher level of carnivory, reflected by a higher percentage of 18:1n9 especially in the small and medium size-classes, was evident in the zooplankton in the northern part of the SoG compared to the Central and South regions. This was further supported by the significant increase of δ^15^N values from south to north. The North differed from the other regions in a greater contribution of green algae (i.e., percentage of 18:3n3) for all size classes of zooplankton. The small size of green algae (nano to pico size range) makes them inaccessible to many zooplankton grazers, requiring an intermediate microzooplankton step in trophic transfer and a longer plankton food chain^59^. This, in turn, would mean that the northern SoG may host a comparatively less efficient food-web in terms of transfer of energy.

We have shown that applying both FA and stable isotope techniques together provides a more complete picture of trophic dynamics. Our study provides evidence that the SoG plankton food-web is largely supported by diatom and mixed flagellate production. However, we also demonstrated a contribution of cyanobacteria, terrestrial material, and green algae (e.g. chlorophyte). The contribution of sources is seasonably variable, with a shift from predominantly diatoms in spring to predominantly mixed flagellates in summer (though diatoms remained important). The seasonal shift in primary producers conferred a higher DHA/EPA ratio to zooplankton in the summer, indicating better quality zooplankton prey for fish during this period. Shifts in plankton phenology, with respect to bloom timing and zooplankton life cycles, may therefore have significant implications for zooplanktivorous fish. Furthermore, this study demonstrated that there is substantial variation in the quality of zooplankton species as prey. Changes in zooplankton species composition, both historically and in the future, are therefore highly relevant to predator nutrition. Future research needs to examine the links between zooplankton and oceanographic conditions in the SoG, to provide a robust framework for the development of a mechanistic understanding of the plankton food webs and the nutritional support that they provide for zooplanktivorous fish.

## Methods

### Study area

**Figure 1:**
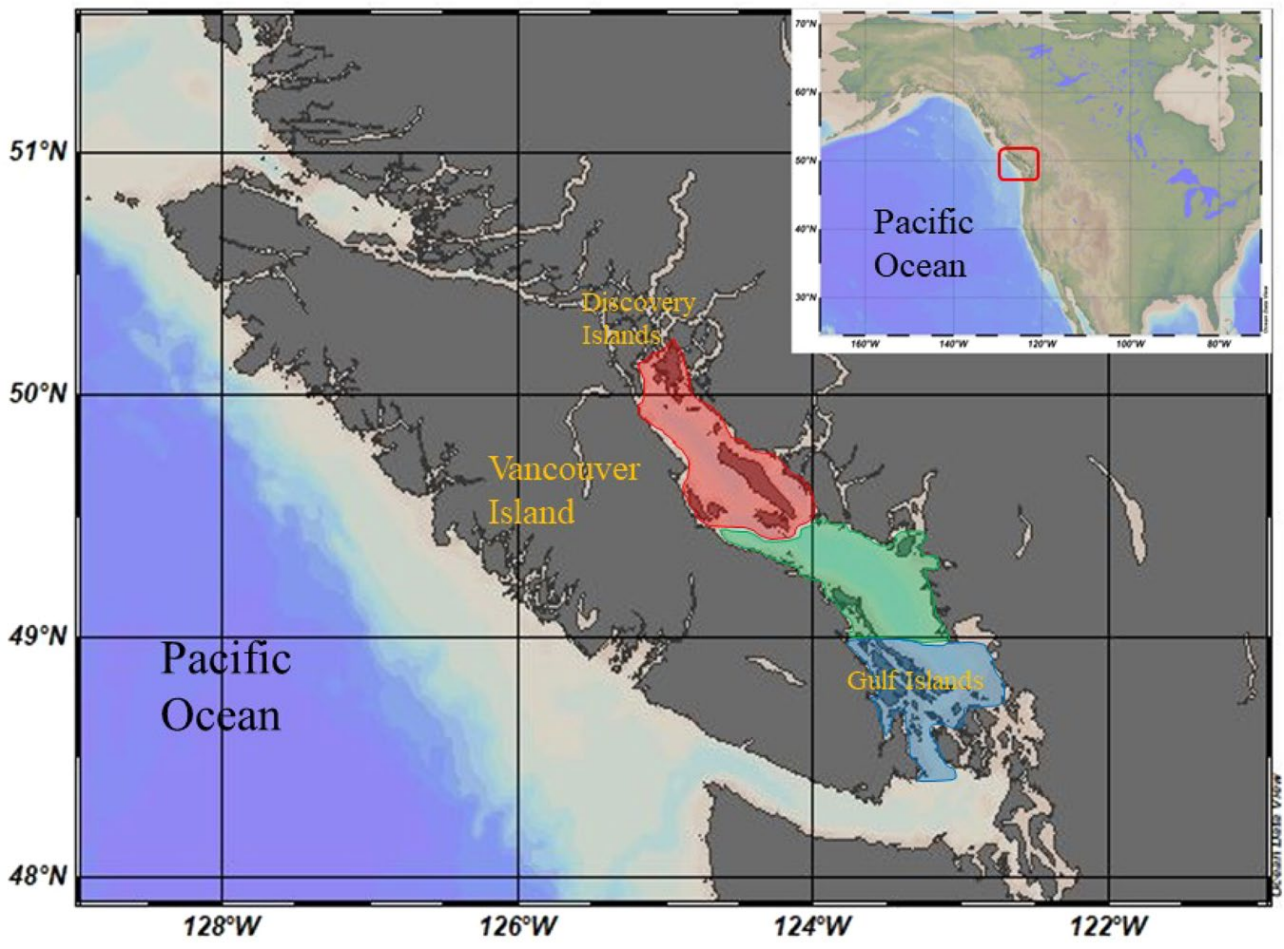
Map of the study region showing the Strait of Georgia with the three regions established for this study (red = north, green = central, blue = south). Figure modified from a map created with Ocean Data View^70^.

The Strait of Georgia (SoG) lies between mainland British Columbia and Vancouver Island and between the Gulf Islands in the south and Discovery Islands in the north (Fig. 1). The Fraser River, which flows into the Central region of the SoG, provides 80% of the freshwater entering the SoG^60^. We grouped samples according to their site of location in three main regions: North (36 sampling stations) - north of 49.48°N, where the influence from the Pacific Ocean and from the Fraser River are weak; Central (36 sampling stations) - between 49.48°N (southern tip of Texada Island) and 49°N (southern part of the Fraser River Delta), which is the area receiving most of the direct influence from the Fraser River; and South (17 sampling stations) - south of 49°N, which comprises the Gulf Islands region (Fig. 1). This sub-regional classification of the SoG closely follows the regionalization by the Department of Fisheries and Oceans (DFO), which is based on hydrographic and plankton characteristics of the region^29^. An annual diatom ‘spring’ phytoplankton bloom generally occurs between March-April^27^. Then, in the early summer months, the water column in the SoG stratifies and the phytoplankton assemblage becomes more flagellate-dominated^34^. The number of samples per region, season and plankton size fraction for FA analyses are detailed in Suppl. Table S3.

**Figure 2:**
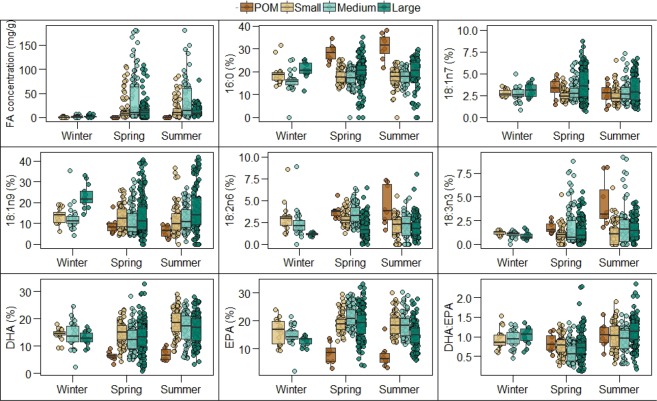
Total fatty acid concentration in mg/g, percentage of selected FAs (16:0, 18:1n7, 18:1n9, 18:2n6, 18:3n3, EPA and DHA) and DHA/EPA of POM and zooplankton size fractions in winter, spring and summer in the Strait of Georgia. The boxes represent the median (black line) and the 25^th^ and 75^th^ percentiles, and the whiskers represent values 1.5 times above/below the interquartiles.

**Figure 3:**
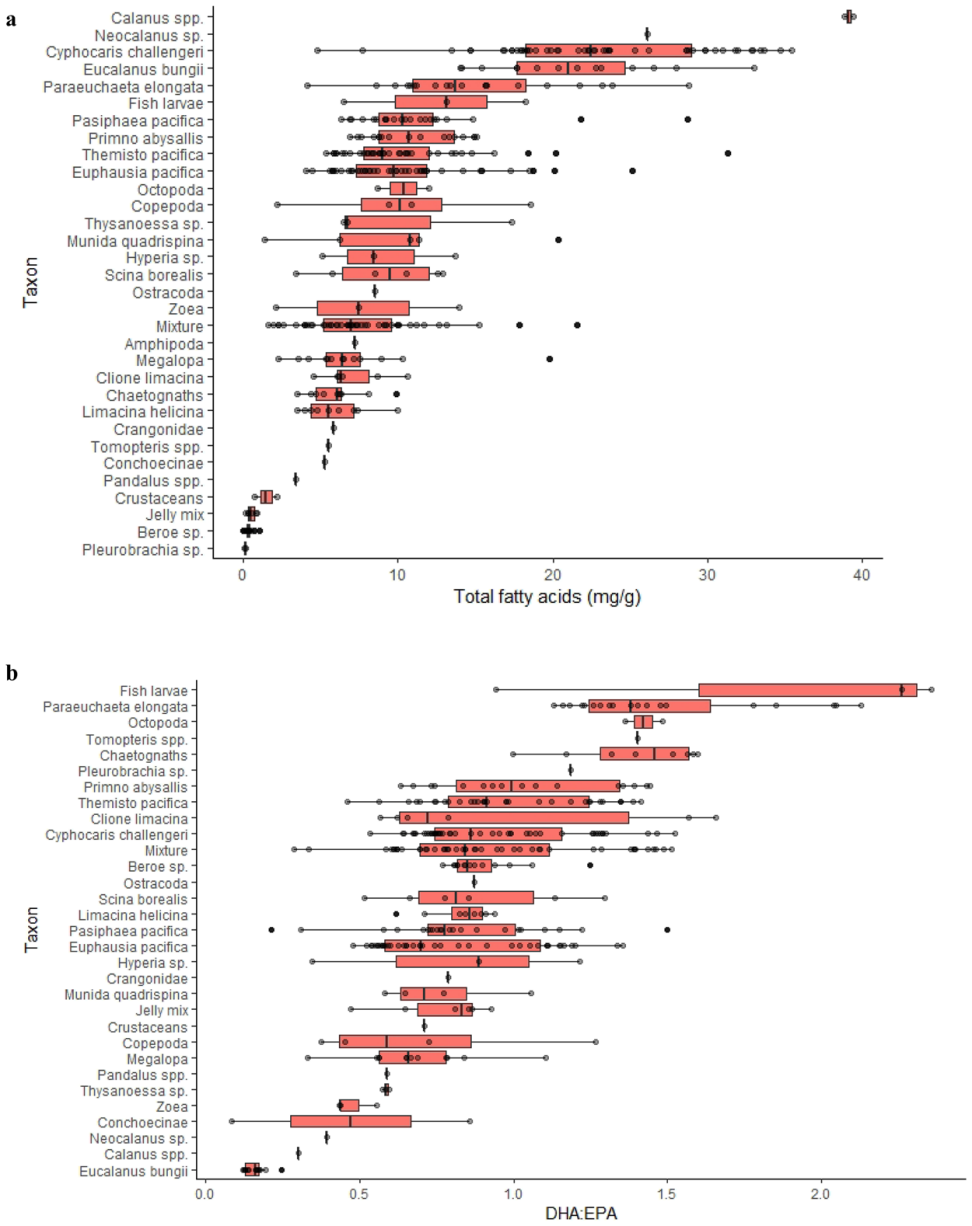
Total fatty acid concentration in mg/g (**a**), and DHA/EPA (**b**) of zooplankton taxa in the North, Central and South regions in spring and summer (combined). The boxes represent the median (black line) and the 25^th^ and 75^th^ percentiles, and the whiskers represent values 1.5 times above/below the interquartiles. The group ‘Mixture’ represents a mix of organisms from taxa that could not be identified to a family level due to its preservation state. In a few cases, it also includes small fractions of crustaceans such as *Cyphocaris* sp., *Euphausia* sp. and decapod larvae as well as fractions of gelatinous plankton.

**Figure 4:**
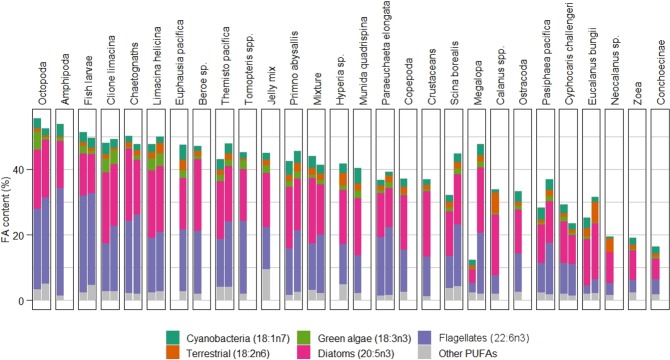
Percentage of selected FA markers and other polyunsaturated FAs (PUFAs; 18:3n6, 20:3n3, 20:3n6, 20:4n6 and 22:5n3) for each large zooplankton species in spring (left column) and summer (right column). The group ‘Mixture’ represents a mix of organisms from taxa that could not be identified to a family level due to its preservation state. In a few cases, it also includes small fractions of crustaceans such as *Cyphocaris* sp, *Euphausia* sp. and decapod larvae as well as fractions of gelatinous plankton.

**Figure 5:**
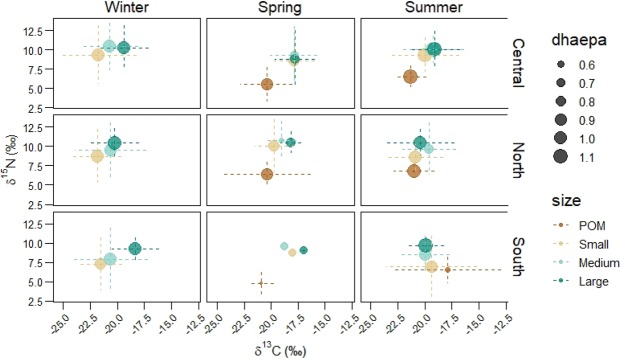
Biplots of δ^13^C and δ^15^N values of POM and zooplankton size classes in winter, summer and spring in the three defined regions in the Strait of Georgia. The colours indicate the plankton size fractions, the size of the circles indicate the value of DHA/EPA, and the dashed lines correspond to the standard errors.

### Sample collection

We collected particulate organic matter (POM) samples in April, May and July 2018 during different cruises through filtration of surface (0–5 m) water on pre-combusted Whatman GF/F filters. We filtered 10 L of water through 47 mm GF/F filters (0.7 µm) and 2 L of water through 25 mm GF/F filters per each sample for FA and isotopic analyses, respectively. POM therefore represented a size class of ~ 0.7 to 100 µm. Zooplankton samples were collected between June 2017 and July 2018 using a 236 µm mesh-size bongo net. The zooplankton net was towed vertically from 10 m above the bottom to surface. Immediately after collection, zooplankton were size-fractionated on board through a set of stacking sieves (4 mm, 2 mm, 1 mm, 500 μm, 250 μm, 125 μm and 64 μm) and flash-frozen in liquid nitrogen.

Juvenile salmon and herring have been reported to feed mostly on large zooplankton^61–64^. Thus, we sorted out individual zooplankton organisms (>1700 µm) from additional samples collected in June-July 2017 and in June, July and September 2018 for species-specific fatty acid and isotopic analyses.

### Fatty acid extraction and quantification

The number of samples analysed from each region and season is specified in Suppl. Table S1. Fatty acid analyses were performed following a one-step fatty acid methyl ester (FAME) method^65^. Wet weights of all samples were measured, the samples were then freeze-dried, and dry weights were measured for calculations of moisture content and to calculate FA concentrations. FAMEs were obtained by lipid extraction in a solution of 2 mL CH_3_OH in 3 N HCl (Sigma-Aldrich cat. #90964-500 ML). Prior to extraction, an internal standard, nonadecanoic acid (19:0), was added following Abdulkadir and Tsuchiya^66^. After extraction, FAME’s were analyzed with a gas chromatograph (Scion 436-GC; Scion Instruments). Peaks were identified against an external standard (Nu-Chek Prep GLC37), measured before and after each sample measurement session.

The specific trophic markers used in this study are detailed in Table 1. For our analyses, we used 18:1n7, 18:1n9, 18:2n6, 18:3n3, EPA, DHA and the ratio of DHA to EPA (DHA/EPA) as proxies for bacteria, carnivory/trophic level, terrestrial/freshwater contribution, green algae, diatoms, flagellates and food quality, respectively. In addition, we included 16:0 in our analyses because of its abundance in all the samples.

### Bulk isotope analysis

A fraction of each frozen zooplankton sample was taken for isotopic analysis, leaving the rest of the sample at -80 °C for FA analysis (Suppl. Table S1). We dried the zooplankton sub-samples in an oven at 60 °C for 48 h and subsequently ground them to a fine powder. In order to remove inorganic carbon, dried zooplankton samples were exposed to concentrated HCl vapour in a desiccator for 4 h at room temperature^67^. The samples were then dried again in the oven (60 °C) for 4–6 h to eliminate vapour excess. We then packed 1.0–1.5 mg of each zooplankton sample into tin capsules. Filters containing POM samples were dried and then packed without weighing. The samples were then sent to the University of Victoria (Victoria, BC, Canada) for bulk C and N isotope analyses, where internal standards were calibrated against the International Atomic Energy Agency (IAEA) standards CH-6 and N-1 in a Europa Scientific 20–20 isotope ratio mass spectrometer (IRMS). δ^13^C and δ^15^N values were determined in parts per thousand (‰) relative to external standards of Vienna Pee Dee Belemnite and atmospheric N_2_.

Stable isotope ratios are expressed following the δ notation:

$$\delta X=\left(\frac{{R}_{sample}}{{R}_{standard}}-1\right)\times {10}^{3}$$

where *X* is ^13^C or ^15^N and *R* the isotopic ratios ^13^C/^12^C or ^15^N/^14^N, respectively. The δ^13^C values were corrected for the effect of lipids both in fish and prey samples following Smyntek *et al*.^68^.

We used the following equation to estimate trophic level (TL):

$$TL=\frac{{{\rm{\delta }}}^{15}{{\rm{N}}}_{consumer}-{{\rm{\delta }}}^{15}{{\rm{N}}}_{baseline}}{3.4}+1$$

where δ^15^N_baseline_ is the mean of the δ^15^N values of POM, and 3.4‰ is assumed to be the ^15^N trophic fractionation factor^15^.

### Data analyses

We grouped zooplankton size fractions as small (64–250 µm), medium (>250–1700 µm) and large (>1700–8000 µm) sizes for data analysis, and we defined seasons as winter (February-March), spring (April-June), and summer (July-September). To explore the relationship between zooplankton size fraction, season and region with respect to FA composition, we performed nonmetric Multidimensional Scaling (nMDS) ordination, which simplifies multivariate data into a few important axes, based on Bray-Curtis dissimilarity matrices. Nonmetric Multidimensional Scaling uses rank orders, and thus is an extremely flexible technique that can accommodate a variety of different kinds of data (e.g. percentage data). To test the effects of the different factors (i.e., region, size -including POM and zooplankton size groups- and season) on the FA composition (in percentage of the total FAs) of POM and zooplankton, we used permutational multivariate analyses of variance (perMANOVA) of the Euclidean distance matrix of the arcsine-square root transformed percent FA dataset (9999 permutations). Then, we performed Tukey’s Honest Significant Difference (HSD) analysis to test for significant differences between groups within each factor.

Next, we used a similarity percentage analysis (SIMPER) test for each matrix to determine the fatty acids that contributed to each grouping (i.e., region, size -including POM and zooplankton size groups- and season). The FAs contributing the most to the differences according to SIMPER were then individually analyzed. In addition, we performed two-way ANOVAs to determine whether there were significant differences between seasons, regions and the interaction between both factors in DHA/EPA of POM and of each zooplankton size class. Nonmetric Multidimensional Scaling, PerMANOVA, Tukey HSD and SIMPER analyses were performed using the package *vegan* in R^69^. ANOVA tests were also performed in R. We used linear regressions of transformed FA percentages to explore the correlations between latitude and the selected FAs.

We tested isotope data for normality with a Shapiro-Wilk’s test. Only δ^13^C values of POM and of zooplankton were normally distributed. Then, we performed Spearman correlation tests to explore the relationship between latitude and both isotopes in POM and all zooplankton size groups. In addition, we performed ANOVAs in R to determine whether there were significant differences between seasons in the δ^13^C and δ^15^N values of POM and of zooplankton size fractions in each region (North, Central and South).

## Supplementary information

Supplemental information.
