# Supplementary material for: Seasonal and spatial dynamics of the planktonic trophic biomarkers in the Strait of Georgia (northeast Pacific) and implications for fish
Source: Sci Rep. 2020 May 22;10:8517. doi: 10.1038/s41598-020-65557-1 (PMC7244518; doi:10.1038/s41598-020-65557-1)

Seasonal and spatial dynamics of the planktonic food-web in the Strait of Georgia (northeast Pacific) and implications for fish.

David Costalago, Ian Forster, Nina Nemcek, Chrys Neville, R. Ian Perry, Kelly Young, Brian P. V. Hunt

Supplementary Table S1. Number of POM and zooplankton size fraction samples used for fatty acid and C and N isotope analyses from each region in the Strait of Georgia and each season.

	Central				North				South			
	POM	Small	Medium	Large	POM	Small	Medium	Large	POM	Small	Medium	Large
Winter	0	8	16	8	0	6	11	5	0	2	4	1
Spring	4	34	43	113	4	36	46	153	1	16	19	28
Summer	5	35	30	87	2	25	21	61	1	20	19	31

Supplementary Table S2. SIMPER results showing only the ten FAs that contributed the most to the differences between seasons (a) and sizes (b).

a)

Spring vs. Summer				Spring vs. Winter				Summer vs. Winter			
	average	sd	cumsum		average	sd	cumsum		average	sd	cumsum
18.1n9	0.0149	0.012	0.0736	18.1n9	0.0143	0.0108	0.0795	20.0	0.0169	0.0091	0.0766
22.6n3	0.0132	0.01	0.1385	20.0	0.0122	0.0081	0.1472	18.1n9	0.0155	0.0124	0.1468
20.1	0.0101	0.008	0.1881	20.5n3	0.0116	0.0087	0.2117	22.6n3	0.0118	0.0087	0.2002
20.5n3	0.0098	0.009	0.2364	14.0	0.0106	0.0085	0.2703	14.0	0.0115	0.0094	0.2521
14.0	0.0097	0.008	0.284	22.6n3	0.0097	0.0077	0.3239	20.5n3	0.0103	0.009	0.2989
20.0	0.009	0.007	0.3284	16.0	0.0087	0.0094	0.3723	16.0	0.0096	0.0107	0.3424
16.0	0.0088	0.01	0.3717	18.2n6	0.008	0.0092	0.4168	18.2n6	0.0095	0.011	0.3853
18.3n3	0.0082	0.006	0.4122	20.3n3	0.0064	0.0067	0.4526	20.1	0.009	0.007	0.426
22.1n9	0.0081	0.008	0.452	16.1	0.0063	0.0055	0.4873	20.3n3	0.0085	0.0077	0.4643
18.2n6	0.0078	0.008	0.4906	24.1	0.0059	0.0053	0.5197	20.4n6	0.0081	0.0046	0.5009

b)

POM vs. Large	average	sd	cumsum	POM vs. Medium	average	sd	cumsum	POM vs. Small	average	sd	cumsum
20:5n3	0.0231	0.0123	0.0833	20:5n3	0.0255	0.0121	0.0895	20:5n3	0.0242	0.0116	0.0885
18:1n9	0.0208	0.0137	0.1585	16:0	0.0218	0.0113	0.1661	22:6n3	0.0219	0.0106	0.1686
18:0	0.0189	0.0159	0.2265	22:6n3	0.0183	0.0115	0.2304	16:0	0.0208	0.0115	0.2445
22:1n9	0.0169	0.0054	0.2874	18:0	0.0181	0.016	0.294	18:0	0.0158	0.0153	0.3026
16:0	0.0144	0.0087	0.3393	22:1n9	0.0157	0.0062	0.3493	18:1n9	0.0156	0.0118	0.3599
22:6n3	0.0137	0.008	0.3887	18:1n9	0.0143	0.0118	0.3997	22:1n9	0.0142	0.0068	0.412
20:0	0.0134	0.0072	0.4372	14:0	0.0126	0.0087	0.444	14:0	0.0125	0.0086	0.4581
14:0	0.0131	0.0083	0.4845	20:0	0.0108	0.0085	0.482	24:1	0.0102	0.0055	0.4957
20:4n6	0.0104	0.0055	0.522	20:1	0.0104	0.0084	0.5186	20:1	0.0098	0.0091	0.5318
20:1	0.0092	0.008	0.555	24:1	0.01	0.0057	0.5539	20:2	0.0097	0.0079	0.5674

Large vs. Medium	average	sd	cumsum	Large vs. Small	average	sd	cumsum	Medium vs. Small	average	sd	cumsum
18:1n9	0.0164	0.01181	0.0907	18:1n9	0.0155	0.0117	0.0851	18:1n9	0.0142	0.0111	0.0765
20:0	0.0104	0.0075	0.1483	22:6n3	0.0107	0.0077	0.1437	22:6n3	0.0114	0.0086	0.1381
22:6n3	0.0104	0.0074	0.2058	20:0	0.0104	0.0068	0.201	14:0	0.0099	0.0081	0.1918
14:0	0.0099	0.0077	0.2608	20:5n3	0.0088	0.0068	0.2493	20:0	0.0097	0.0074	0.2439
20:5n3	0.0096	0.0076	0.3141	20:4n6	0.0082	0.0052	0.2944	20:1	0.0088	0.0078	0.2916
16:0	0.0087	0.0077	0.3626	16:0	0.0082	0.008	0.3394	20:5n3	0.008	0.0064	0.3347
18:2n6	0.0078	0.0077	0.4061	24:1	0.0081	0.005	0.3838	18:3n3	0.0078	0.006	0.377
24:1	0.0078	0.0052	0.4494	18:3n3	0.0074	0.0057	0.4244	16:0	0.0074	0.0087	0.4171
20:1	0.0077	0.0072	0.4922	14:0	0.0072	0.0059	0.4643	18:2n6	0.0072	0.0084	0.4561
20:4n6	0.0076	0.0054	0.5345	18:2n6	0.0066	0.006	0.5004	22:1n9	0.0069	0.0077	0.4934

Supplementary Table S3. Mean ± SE (n) of total FA concentration, of percentage of the selected FAs and of DHA/EPA in POM and zooplankton size fraction samples in the three Strait of Georgia regions in winter, spring and summer

Total FA in mg/g.	Winter			Spring			Summer		
	North	Central	South	North	Central	South	North	Central	South
POM	NA	NA	NA	0.18±0.01 (4)	0.17±0.03 (6)	0.14±0.05 (2)	0.33±0.02 (2)	0.32±0.04 (5)	0.21 (1)
Small	0.77±0.32 (6)	0.59±0.26 (8)	0.18±0.06 (2)	9.06±2.09 (33)	14.02±3.78 (32)	26.62±7.76 (15)	18.89±4.14 (20)	14.68±3.53 (33)	18.74±4.48 (16)
Medium	2.04±0.70 (9)	1.73±0.59 (14)	0.07±0.02 (3)	15.97±3.51 (31)	19.25±4.40 (39)	37.39±7.74 (18)	24.39±7.45 (16)	22.09±5.13 (28)	31.33±7.28 (16)
Large	3.34±1.24 (5)	1.64±0.92 (8)	NA	14.12±1.23 (134)	9.55±1.18 (88)	5.37±1.37 (21)	14.75±1.23 (55)	11.56±0.83 (81)	9.95±1.73 (25)

% 16:0	Winter			Spring			Summer		
	North	Central	South	North	Central	South	North	Central	South
POM	NA	NA	NA	28.82±4.23 (4)	31.06±1.34 (6)	32.56±5.83 (2)	29.47±0.55 (2)	33.68±2.23 (5)	21.57 (1)
Small	17.66±1.05 (6)	18.63±1.51 (8)	25.75±5.75 (2)	17.07±0.53 (33)	18.39±0.49 (32)	19.09±0.92 (15)	15.81±1.23 (20)	17.51±0.49 (33)	18.08±1.14 (16)
Medium	13.24±1.52 (9)	16.58±0.80 (14)	21.58±1.83 (3)	16.33±0.69 (31)	17.38±0.50 (39)	17.95±0.47 (18)	16.14±0.89 (16)	17.80±0.41 (28)	18.46±0.88 (16)
Large	21.60±1.19 (5)	21.35±1.51 (8)	NA	20.17±0.45 (134)	20.31±0.50 (88)	20.30±0.92 (21)	20.49±0.90 (55)	19.61±0.64 (81)	20.25±0.62 (25)

% 18:1n7	Winter			Spring			Summer		
	North	Central	South	North	Central	South	North	Central	South
POM	NA	NA	NA	2.75±0.65 (4)	2.84±0.58 (6)	2.41±1.09 (2)	3.87±0.53 (2)	2.35±0.53 (5)	2.27 (1)
Small	2.81±0.13 (6)	2.90±0.17 (8)	2.44±0.02 (2)	2.48±0.09 (33)	2.59±0.11 (32)	2.45±0.11 (15)	5.20±1.81 (20)	2.47±0.19 (33)	2.26±0.19 (16)
Medium	2.57±0.21 (9)	2.64±0.16 (14)	2.86±0.33 (3)	2.68±0.11 (31)	2.78±0.12 (39)	3.11±0.76 (18)	3.66±0.91 (16)	2.57±0.12 (28)	3.41±0.39 (16)
Large	2.81±0.25 (5)	3.36±0.28 (8)	NA	4.68±0.37 (134)	3.82±0.18 (88)	3.43±0.37 (21)	7.26±1.45 (55)	3.71±0.40 (81)	3.04±0.32 (25)

% 18:1n9	Winter			Spring			Summer		
	North	Central	South	North	Central	South	North	Central	South
POM	NA	NA	NA	8.21±3.26 (4)	6.91±0.98 (6)	8.00±1.78 (2)	7.26±1.19 (2)	6.15±1.55 (5)	4.43 (1)
Small	14.07±1.15 (6)	14.33±1.24 (8)	8.04±1.97 (2)	15.25±1.00 (33)	12.74±0.77 (32)	9.50±1.31 (15)	12.05±1.44 (20)	12.34±1.20 (33)	10.38±2.44 (16)
Medium	12.32±1.13 (9)	11.79±1.01 (14)	6.89±1.50 (3)	11.59±1.14 (31)	12.03±1.68 (39)	8.75±0.86 (18)	13.72±1.84 (16)	13.55±1.08 (28)	9.61±1.11 (16)
Large	23.99±2.64 (5)	22.25±1.96 (8)	NA	14.15±0.78 (134)	14.26±0.95 (88)	8.67±1.23 (21)	15.35±1.57 (55)	18.29±1.17 (81)	16.86±1.89 (25)

% 18:2n6	Winter			Spring			Summer		
	North	Central	South	North	Central	South	North	Central	South
POM	NA	NA	NA	3.89±0.90 (4)	3.17±0.24 (6)	3.15±0.66 (2)	3.08±0.56 (2)	4.61±1.15 (5)	6.70 (1)

Small	2.65±0.54 (6)	3.40±0.78 (8)	2.84±0.99 (2)	2.74±0.12 (33)	2.68±0.11 (32)	3.47±0.16 (15)	2.08±0.25 (20)	2.02±0.19 (33)	2.11±0.33 (16)
Medium	2.52±0.28 (9)	2.53±0.53 (14)	1.10±0.73 (3)	3.70±0.16 (31)	3.15±0.19 (39)	2.90±0.17 (18)	2.15±0.35 (16)	2.07±0.25 (28)	1.88±0.32 (16)
Large	1.21±0.07 (5)	1.08±0.04 (8)	NA	1.82±0.08 (134)	1.86±0.06 (88)	3.08±0.55 (21)	2.07±0.16 (55)	1.96±0.12 (81)	1.69±0.15 (25)

**% 18:3n3**	Winter			Spring			Summer		
	North	Central	South	North	Central	South	North	Central	South
POM	NA	NA	NA	0.43±0.19 (4)	0.69±0.09 (6)	1.24±0.02 (2)	0.23±0.23 (2)	0.30±0.12 (5)	0 (1)
Small	0.31±0.07 (6)	0.38±0.09 (8)	0.21±0.04 (2)	0.88±0.11 (33)	0.71±0.14 (32)	0.81±0.22 (15)	1.33±0.27 (20)	1.19±0.18 (33)	0.86±2.27 (16)
Medium	0.15±0.03 (9)	0.18±0.04 (14)	0.43±0.33 (3)	0.42±0.06 (31)	0.43±0.08 (39)	0.42±0.09 (18)	0.91±0.24 (16)	0.75±0.15 (28)	0.40±0.14 (16)
Large	0.14±0.02 (5)	0.88±0.11 (8)	NA	0.61±0.05 (134)	0.38±0.03 (88)	0.59±0.12 (21)	0.17±0.01 (55)	0.25±0.03 (81)	0.37±0.07 (25)

**% DHA**	Winter			Spring			Summer		
	North	Central	South	North	Central	South	North	Central	South
POM	NA	NA	NA	5.82±1.16 (4)	5.61±0.81 (6)	4.16±2.48 (2)	6.68±1.88 (2)	6.67±1.05 (5)	9.42 (1)
Small	14.37±0.51 (6)	14.14±1.12 (8)	14.68±0.38 (2)	15.05±0.69 (33)	15.36±0.72 (32)	14.08±1.04 (15)	15.95±1.01 (20)	19.11±0.91 (33)	18.6±1.36 (16)
Medium	13.63±1.24 (9)	14.14±0.93 (14)	19.57±3.44 (3)	10.85±0.57 (31)	13.72±0.82 (39)	15.87±0.62 (18)	11.83±1.26 (16)	18.75±0.90 (18)	19.50±0.68 (16)
Large	12.85±1.18 (5)	12.97±0.90 (8)	NA	13.37±0.42 (134)	14.18±0.42 (88)	13.50±1.53 (21)	13.59±0.70 (55)	16.51±0.58 (81)	18.35±0.99 (25)

**% EPA**	Winter			Spring			Summer		
	North	Central	South	North	Central	South	North	Central	South
POM	NA	NA	NA	6.88±1.63 (4)	6.81±1.59 (6)	7.98±4.81 (2)	6.25±0.75 (2)	6.14±1.13 (5)	17.06 (1)
Small	15.82±1.65 (6)	15.61±1.65 (8)	18.97±4.99 (2)	17.91±0.36 (33)	18.67±0.35 (32)	22.39±1.25 (15)	17.23±0.77 (20)	17.99±0.59 (33)	21.52±1.19 (16)
Medium	14.84±0.73 (9)	14.39±0.49 (14)	19.71±0.91 (3)	19.99±0.62 (31)	20.51±0.40 (39)	25.74±0.71 (18)	16.56±0.94 (16)	16.94±0.60 (28)	22.85±1.20 (16)
Large	12.19±0.79 (5)	13.24±0.58 (8)	NA	18.54±0.34 (134)	19.58±0.53 (88)	22.01±1.55 (21)	3.95±0.53 (55)	15.19±0.51 (81)	17.08±1.08 (25)

**DHA/EPA**	Winter			Spring			Summer		
	North	Central	South	North	Central	South	North	Central	South
POM	NA	NA	NA	0.90±0.08 (4)	0.91±0.09 (6)	0.52±0.00 (2)	1.04±0.17 (2)	1.14±0.13 (5)	0.55 (1)
Small	0.97±0.12 (6)	0.95±0.09 (8)	0.82±0.19 (2)	0.85±0.04 (33)	0.82±0.04 (32)	0.64±0.04 (15)	0.95±0.06 (20)	1.10±0.06 (33)	0.93±0.10 (16)
Medium	0.91±0.06 (9)	0.97±0.05 (14)	0.99±0.18 (3)	0.56±0.03 (31)	0.68±0.04 (39)	0.63±0.03 (18)	0.72±0.06 (16)	1.11±0.04 (28)	0.89±0.06 (16)
Large	1.07±0.11 (5)	0.99±0.07 (8)	NA	0.75±0.03 (134)	0.74±0.02 (88)	0.63±0.08 (21)	0.99±0.05 (55)	1.14±0.04 (81)	1.13±0.06 (25)

Supplementary Table S4. Mean ± SE (n) of C and N isotopic values and trophic level in POM and zooplankton size fraction samples, and mean trophic level of analysed zooplankton taxa in the three Strait of Georgia regions.

**δ^13^C** of POM and zooplankton size fractions	Winter			Spring			Summer		
	North	Central	South	North	Central	South	North	Central	South
POM	NA	NA	NA	-20.38±2.07 (8)	-20.41±1.23 (12)	-20.89±0.58 (6)	-20.98±0.97 (5)	-21.28±0.74 (6)	-17.88±2.53 (4)
Small	-21.83±1.12 (12)	-21.86±1.84 (18)	-21.57±1.04 (5)	-19.34±0.84 (22)	-18.25±1.19 (35)	-20.11±1.32 (5)	-21.12±1.34 (20)	-20.19±1.68 (40)	-19.36±2.34 (19)
Medium	-20.99±1.90 (19)	-20.73±1.63 (27)	-20.85±1.81 (10)	-18.86±0.77 (28)	-18.13±1.01 (46)	-19.09±0.45 (5)	-19.7±1.32 (17)	-19.25±1.48 (39)	-19.95±1.17 (19)
Large	-20.24±1.15 (15)	-19.37±1.16 (29)	-19.26±2.09 (4)	-18.55±0.63 (16)	-17.87±0.92 (46)	-17.51±0.93 (3)	-20.4±1.61 (5)	-19.05±1.2 (38)	-19.86±0.86 (4)

**δ^15^N** of POM and zooplankton size fractions	Winter			Spring			Summer		
	North	Central	South	North	Central	South	North	Central	South
POM	NA	NA	NA	6.37±0.82 (8)	5.51±1.13 (12)	4.73±0.73 (6)	6.82±0.49 (5)	6.52±0.7 (6)	6.49±0.87 (4)
Small	8.73±1.76 (12)	9.08±2.12 (18)	7.24±1.75 (5)	9.82±1.11 (22)	8.87±1.38 (35)	8.13±0.74 (5)	7.01±3.69 (20)	8.49±2.91 (40)	5.66±3.1 (19)
Medium	8.25±3.49 (19)	10.38±1.55 (27)	5.17±3.45 (10)	10.35±0.96 (28)	9.68±1.64 (46)	8.11±1.44 (5)	9.3±2.19 (17)	10.02±0.91 (39)	8.48±1.21 (19)
Large	10.45±0.93 (15)	9.958±2.13 (29)	7.88±2.73 (4)	10.07±0.67 (16)	9.01±1.81 (46)	9.22±0.41 (3)	10.5±0.86 (5)	10.01±1.194 (38)	9.67±0.49 (4)

**Trophic Level** of POM and zooplankton size fractions.	Spring			Summer		
	North	Central	South	North	Central	South
POM	NA	NA	NA	NA	NA	NA
Small	1.79±0.32 (22)	1.99±0.40 (35)	2±0.21 (5)	1.05±1.08 (20)	1.58±0.85 (40)	0.75±0.91 (19)
Medium	1.95±0.28 (28)	2.23±0.48 (46)	1.99±0.42 (5)	1.73±0.64 (17)	2.03±0.27 (39)	1.58±0.35 (19)
Large	1.87±0.19 (16)	2.03±0.53 (46)	2.31±0.12 (3)	2.08±0.25 (5)	2.02±0.35 (38)	1.93±0.14 (4)

**Mean Trophic Level** of zooplankton taxa.	**Spring**			**Summer**		
	Central	North	South	Central	North	South
Beroe sp.	2.5	1.85	2.55			
Chaetognaths	3.25			2.85	2.77	2.92
Clione limacina				1.87		
Cyphocaris challengeri				2.28	2.19	
Eucalanus bungii				1.59		
Euphausia pacifica	2.1			1.67	1.52	
Fish larvae				2.91	2.62	
Megalopa				2.03		1.99
Munidae				2.01	2.11	

Mysids					1.33
Pasiphaea pacifica	2.8		2.08	1.97	
Primno abysallis	2.49		2.76	1.96	
Scina borealis			2.96	2.54	
Themisto pacifica		2.25		2.94	
Zoea		1.08			0.19

Supplementary figure S1. Nonmetric Multidimensional Scaling (nMDS) ordination plots of percentage of FAs (arcsine transformed) of zooplankton samples ordinated by Strait of Georgia region (A), season (B) and zooplankton size class (C)

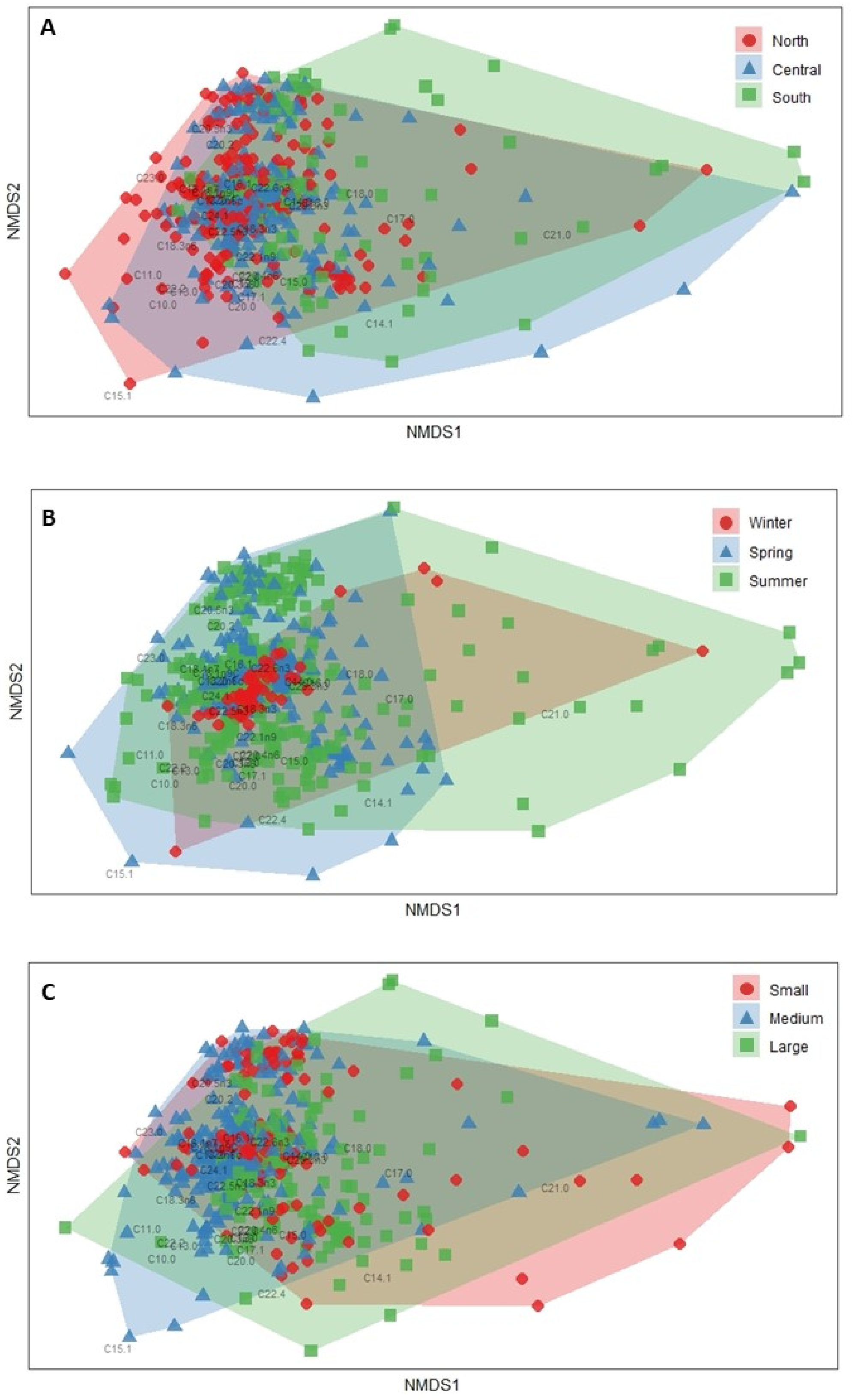

Supplementary figure S2. Total fatty acid concentration (in mg/g) and percentages of selected FA (16:0, 18:1n7, 18:1n9, 18:2n6, 18:3n3, EPA and DHA) and DHA/EPA in relation to latitude for each plankton size group in the Strait of Georgia (SoG). Colours correspond to the three defined SoG regions.

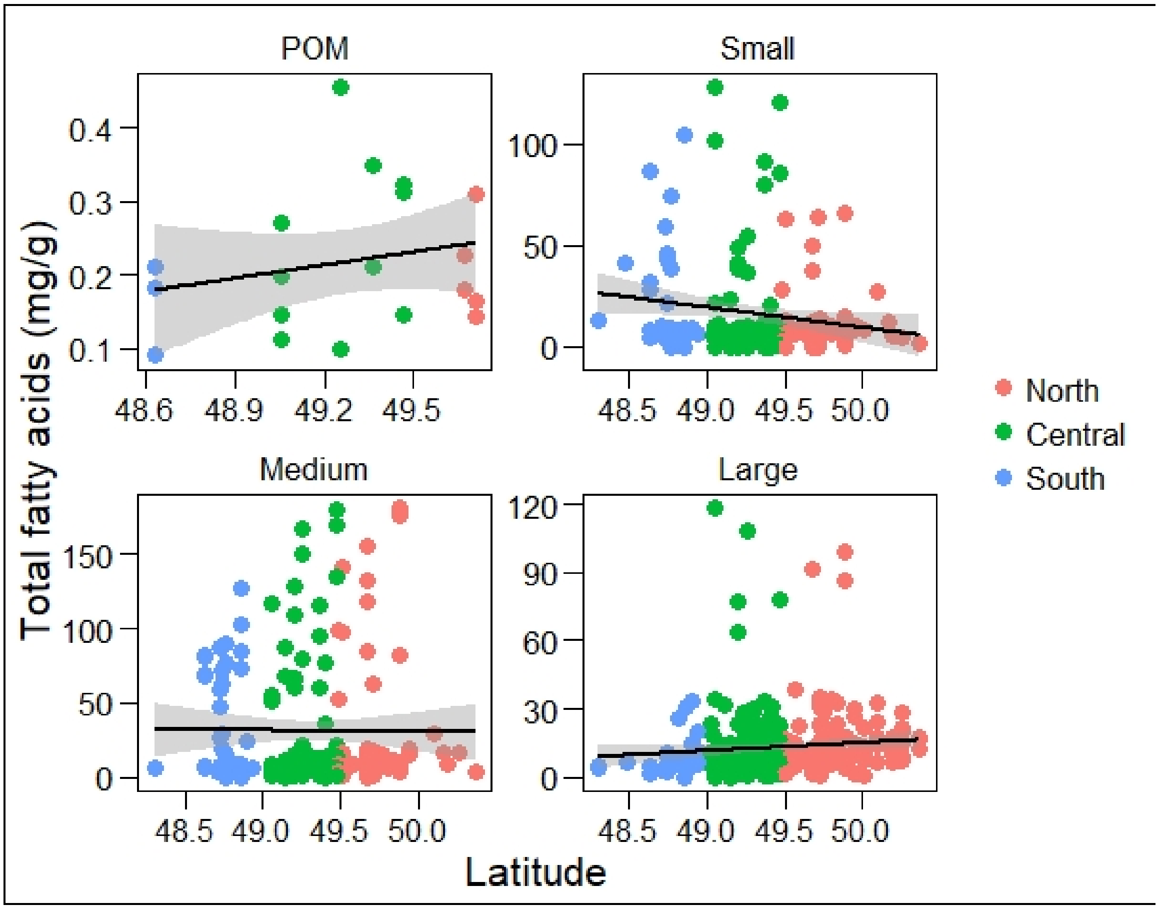

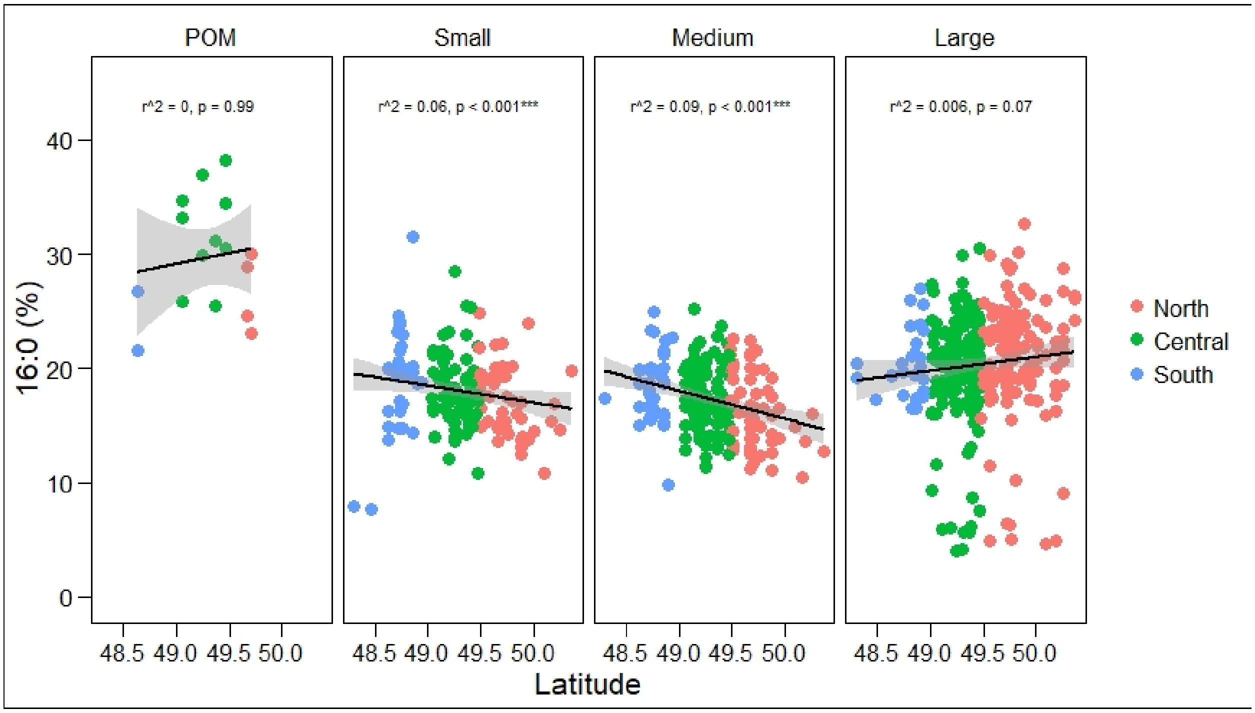

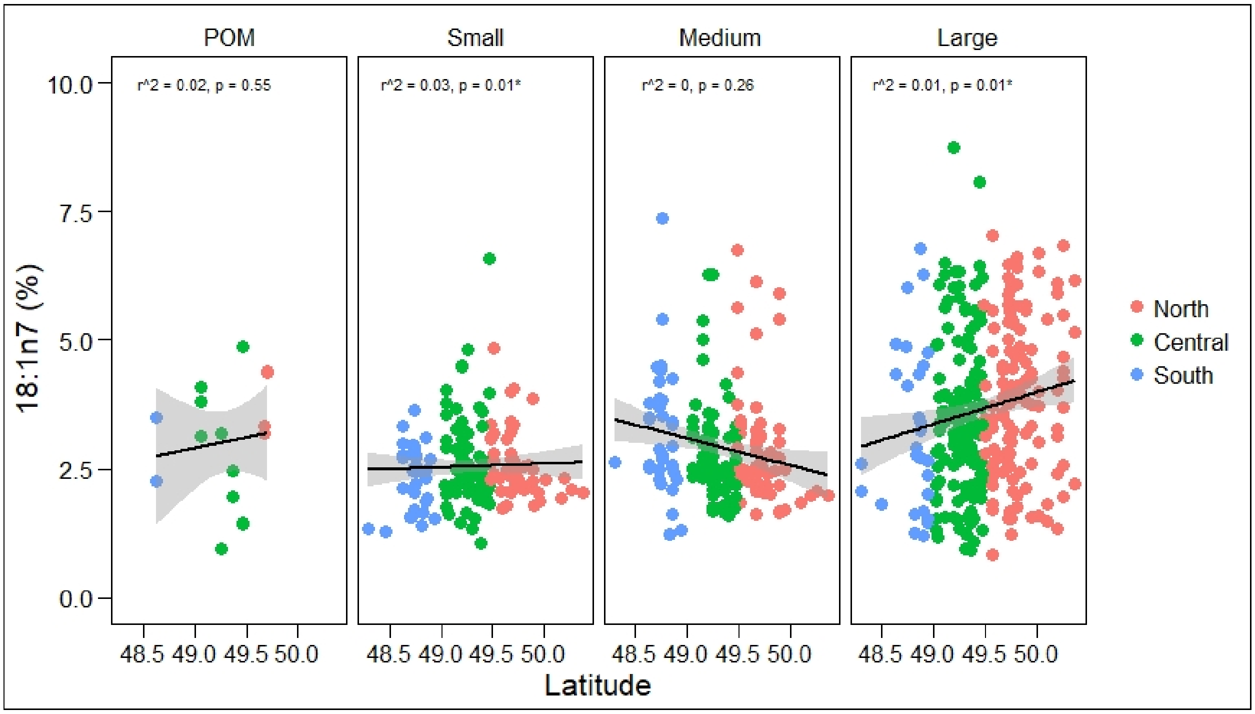
POM
Small
Medium
Large
r^2 = 0.02, p = 0.55
r^2 = 0.03, p = 0.01*
r^2 = 0, p = 0.26
r^2 = 0.01, p = 0.01*
18:1n7 (%)
10.0
7.5
5.0
2.5
0.0
48.5 49.0 49.5 50.0
Latitude
North
Central
South

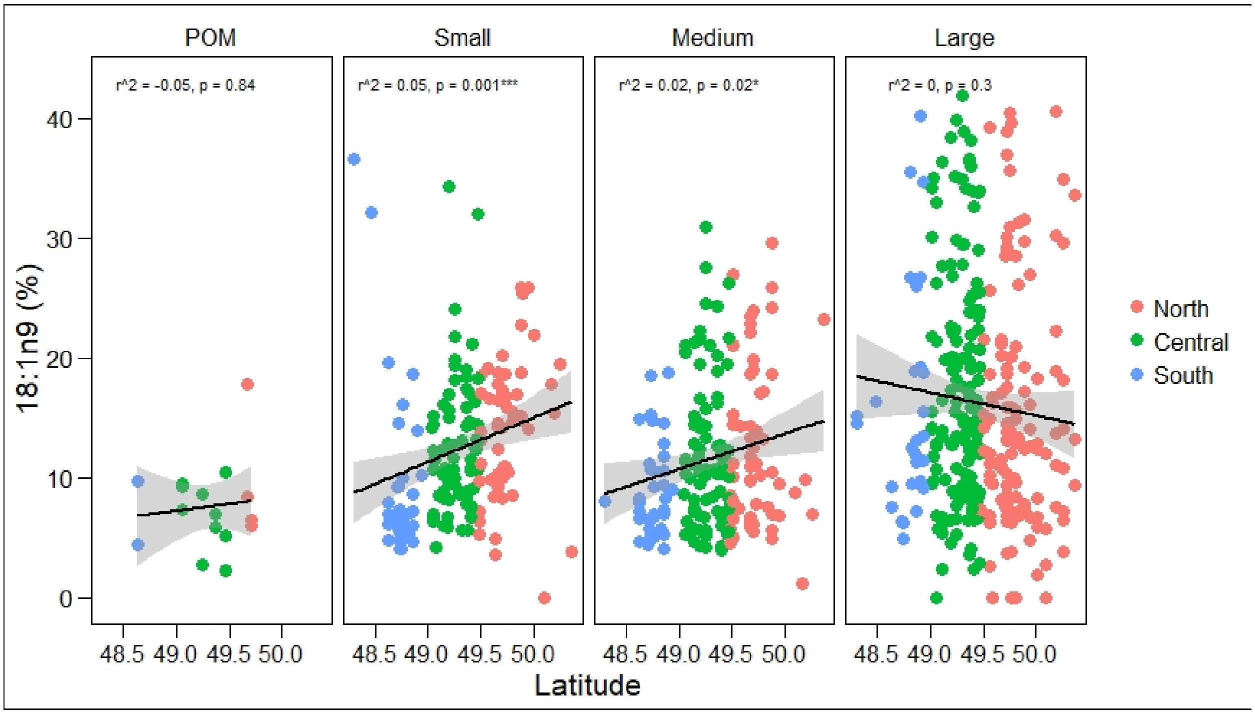
POM
Small
Medium
Large
r^2 = -0.05, p = 0.84
r^2 = 0.05, p = 0.001***
r^2 = 0.02, p = 0.02*
r^2 = 0, p = 0.3
18:1n9 (%)
40
30
20
10
0
48.5 49.0 49.5 50.0
Latitude
North
Central
South

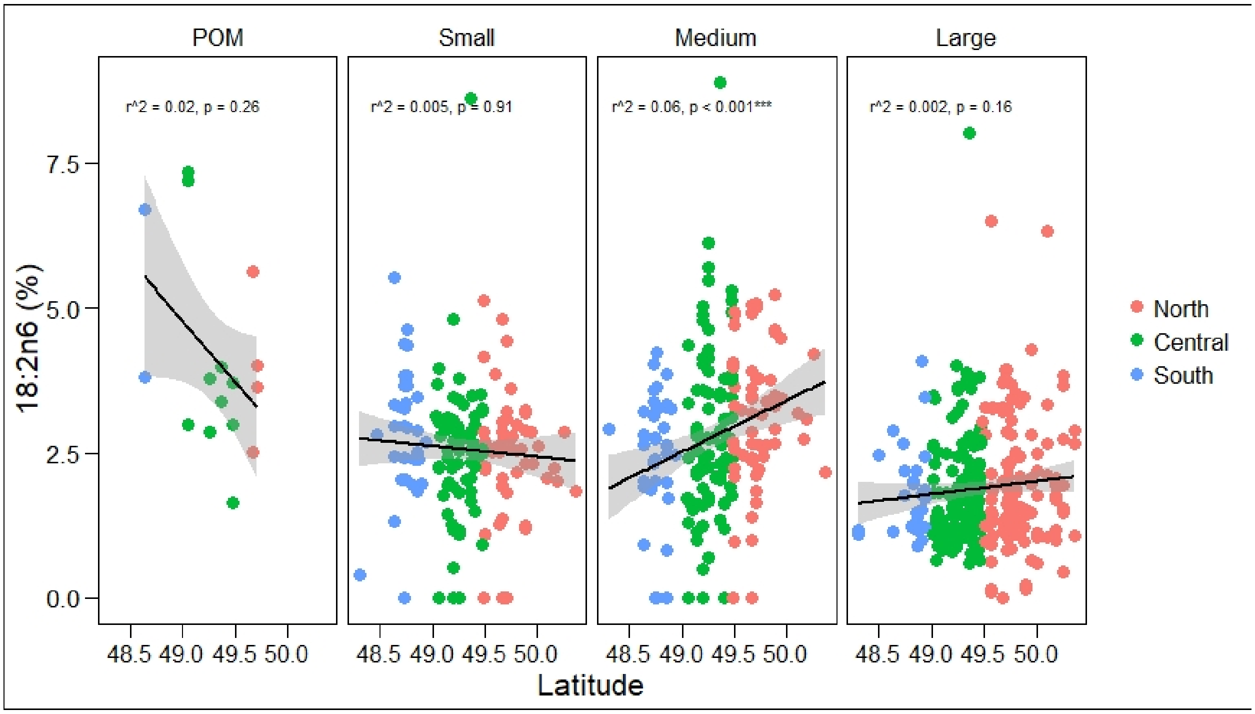
POM
Small
Medium
Large
r^2 = 0.02, p = 0.26
r^2 = 0.005, p = 0.91
r^2 = 0.06, p < 0.001***
r^2 = 0.002, p = 0.16
18:2n6 (%)
0.0
2.5
5.0
7.5
48.5 49.0 49.5 50.0
Latitude
North
Central
South

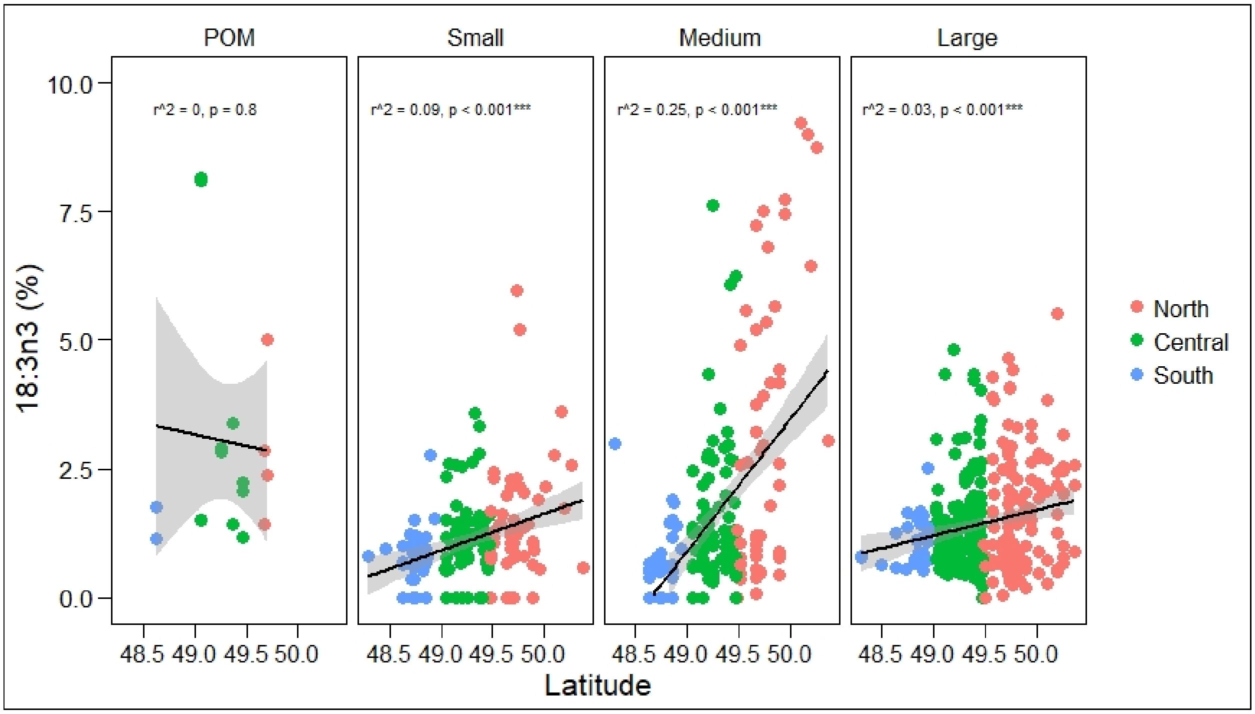
POM
Small
Medium
Large
r^2 = 0, p = 0.8
r^2 = 0.09, p < 0.001***
r^2 = 0.25, p < 0.001***
r^2 = 0.03, p < 0.001***
18:3n3 (%)
0.0
2.5
5.0
7.5
10.0
48.5 49.0 49.5 50.0
Latitude
North
Central
South

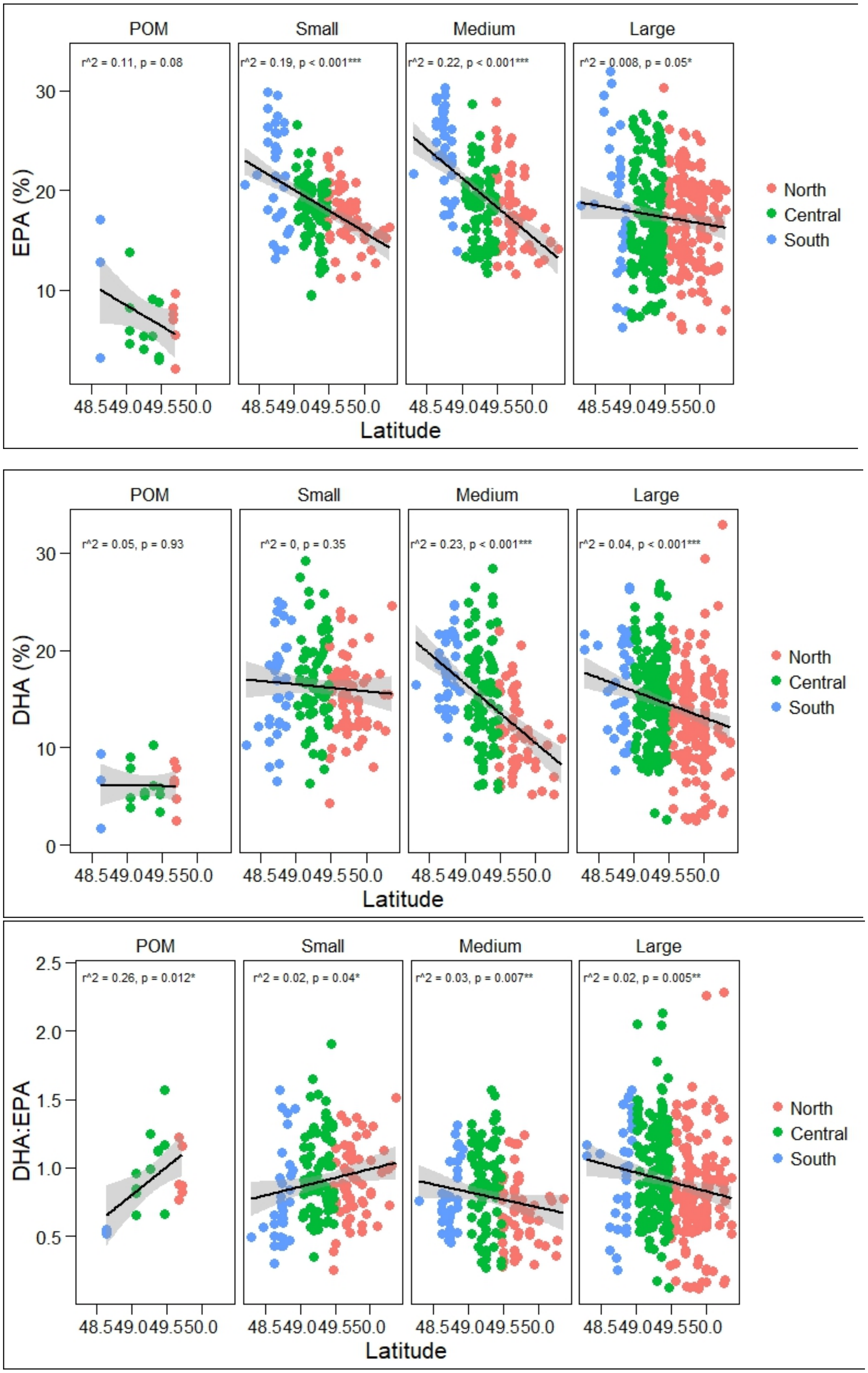
POM
Small
Medium
Large
r^2 = 0.11, p = 0.08
r^2 = 0.19, p < 0.001***
r^2 = 0.22, p < 0.001***
r^2 = 0.008, p = 0.05*
EPA (%)
Latitude
North
Central
South
r^2 = 0.05, p = 0.93
r^2 = 0, p = 0.35
r^2 = 0.23, p < 0.001***
r^2 = 0.04, p < 0.001***
DHA (%)
r^2 = 0.26, p = 0.012*
r^2 = 0.02, p = 0.04*
r^2 = 0.03, p = 0.007**
r^2 = 0.02, p = 0.005**
DHA:EPA

Supplementary figure S3. Percentage of selected FAs (16:0, 18:1n7, 18:1n9, 18:2n6, 18:3n3, EPA and DHA) of zooplankton species in the Strait of Georgia. The boxes represent the median and the 25th and 75th percentiles, the whiskers represent values 1.5 times above/below the interquartiles, and black points are outliers.

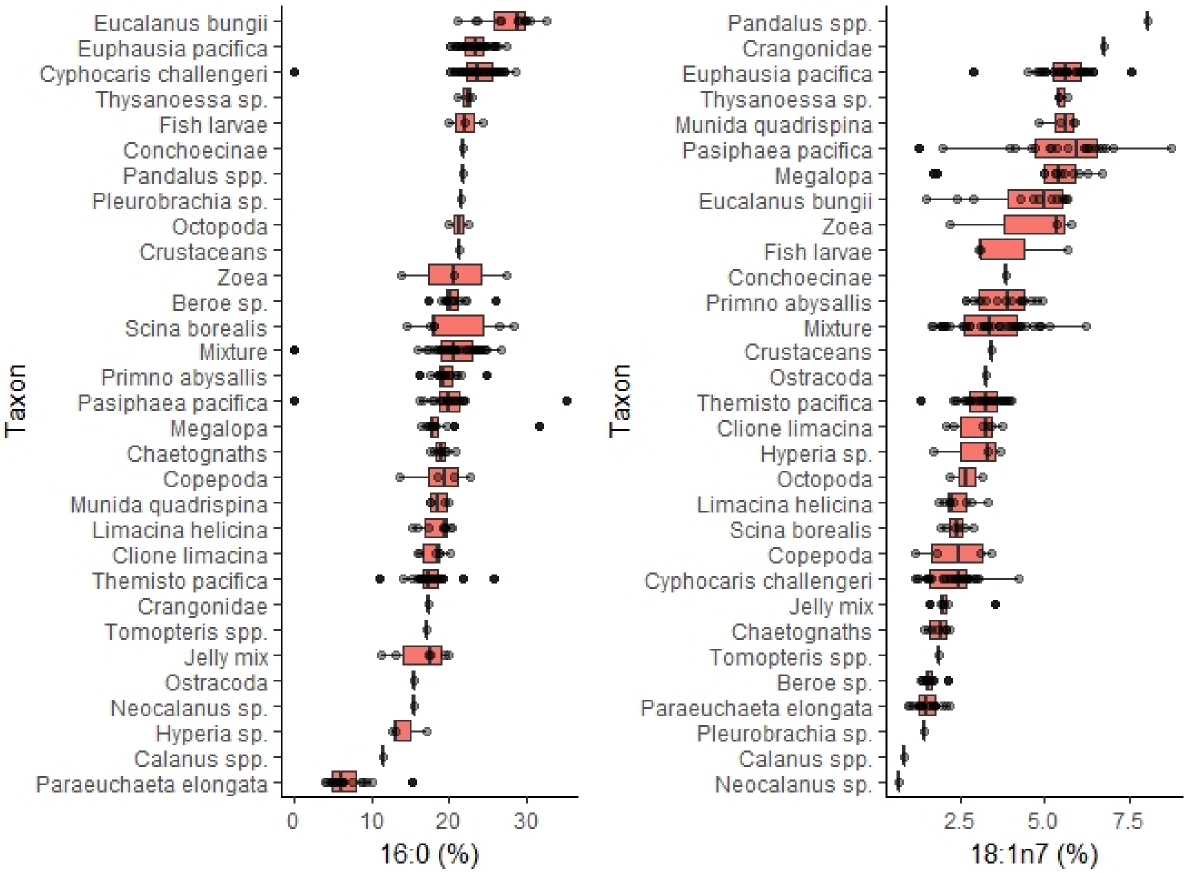

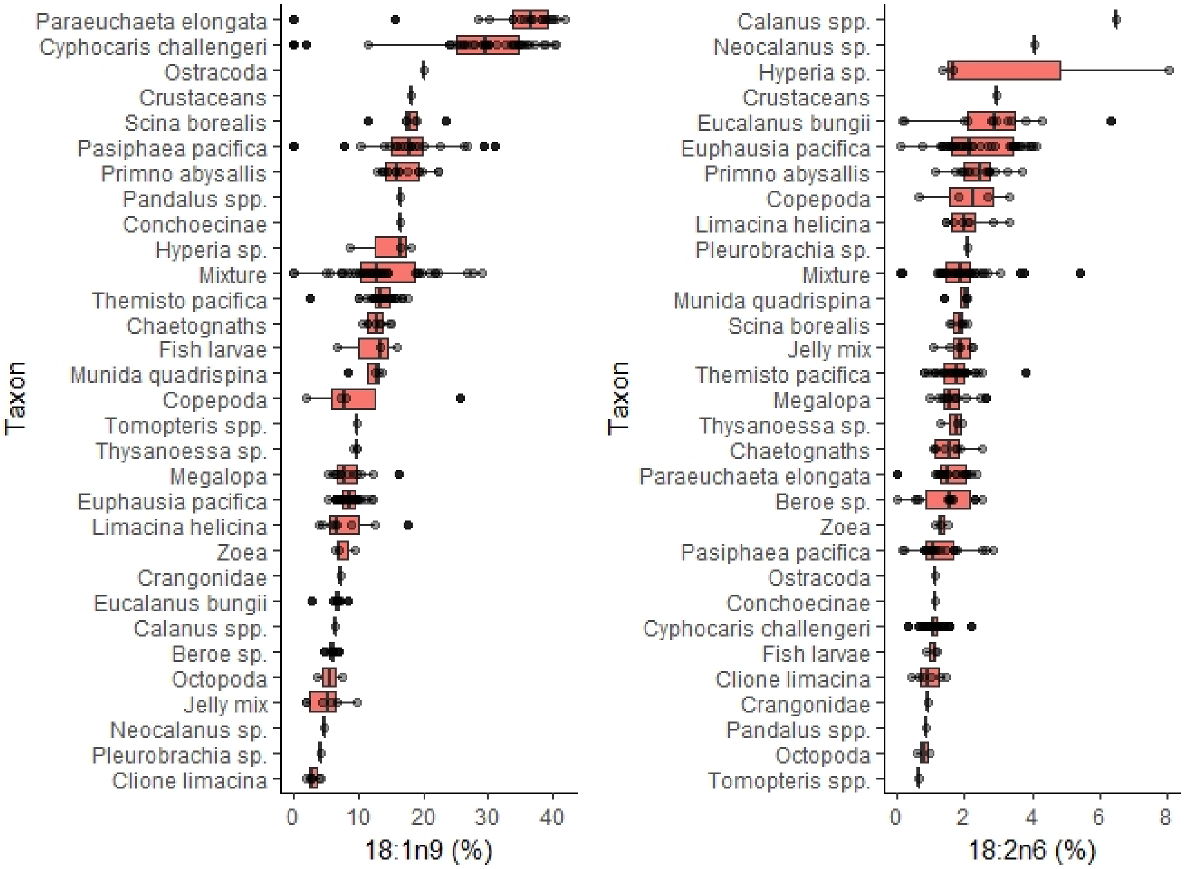

Paraeuchaeta elongata
Cyphocaris challengeri
Ostracoda
Crustaceans
Scina borealis
Pasiphaea pacifica
Primno abysallis
Pandalus spp.
Conchoecinae
Hyperia sp.
Mixture
Themisto pacifica
Chaetognaths
Fish larvae
Munida quadrispina
Copepoda
Tomopteris spp.
Thysanoessa sp.
Megalopa
Euphausia pacifica
Limacina helicina
Zoea
Crangonidae
Eucalanus bungii
Calanus spp.
Beroe sp.
Octopoda
Jelly mix
Neocalanus sp.
Pleurobrachia sp.
Clione limacina
Taxon
0
10
20
30
40
18:1n9 (%)
Calanus spp.
Neocalanus sp.
Hyperia sp.
Crustaceans
Eucalanus bungii
Euphausia pacifica
Primno abysallis
Copepoda
Limacina helicina
Pleurobrachia sp.
Mixture
Munida quadrispina
Scina borealis
Jelly mix
Themisto pacifica
Megalopa
Thysanoessa sp.
Chaetognaths
Paraeuchaeta elongata
Beroe sp.
Zoea
Pasiphaea pacifica
Ostracoda
Conchoecinae
Cyphocaris challengeri
Fish larvae
Clione limacina
Crangonidae
Pandalus spp.
Octopoda
Tomopteris spp.
Taxon
0
2
4
6
8
18:2n6 (%)

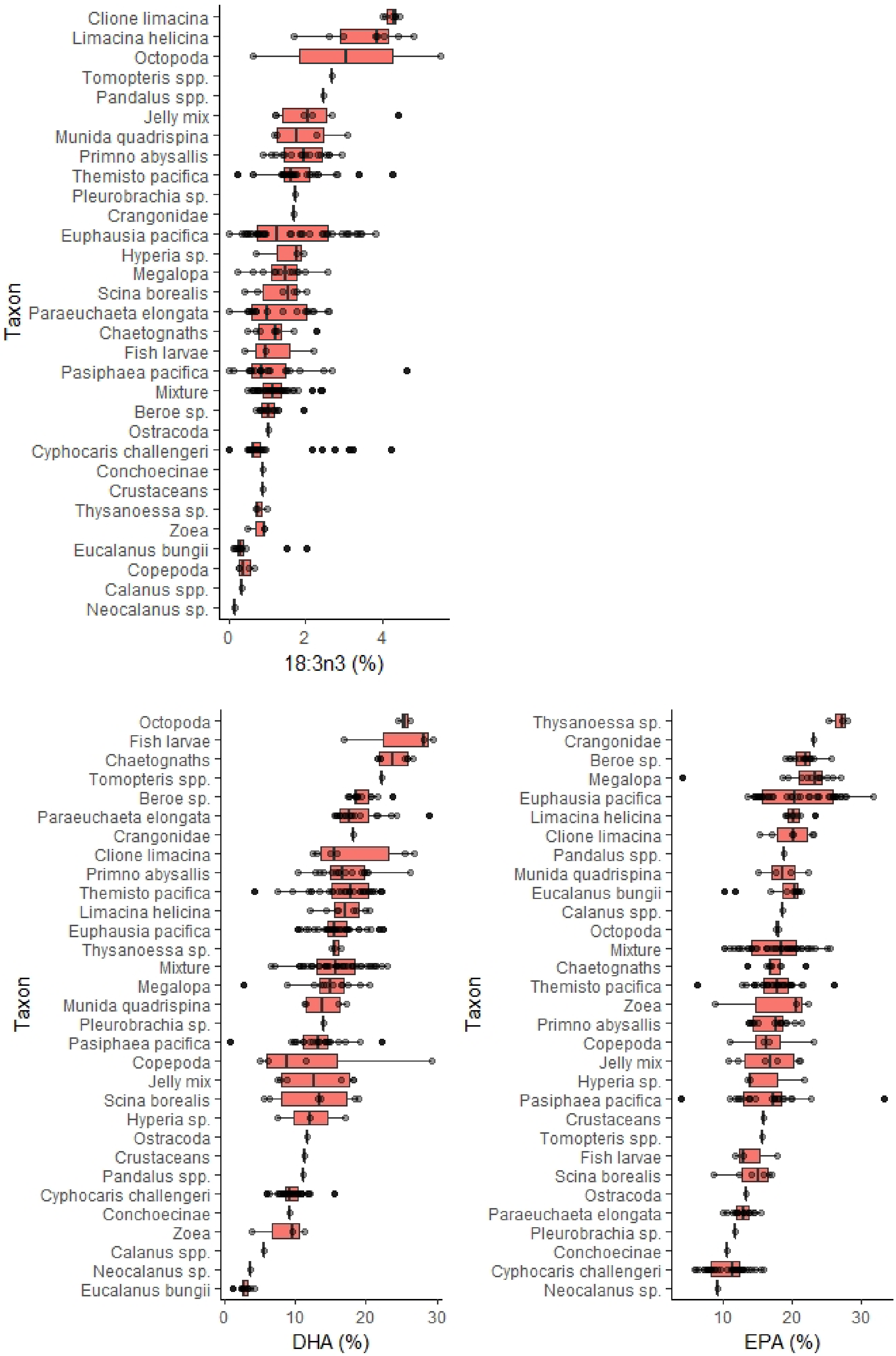
Clione limacina
Limacina helicina
Octopoda
Tomopteris spp.
Pandalus spp.
Jelly mix
Munida quadrispina
Primno abysallis
Themisto pacifica
Pleurobrachia sp.
Crangonidae
Euphausia pacifica
Hyperia sp.
Megalopa
Scina borealis
Paraeuchaeta elongata
Chaetognaths
Fish larvae
Pasiphaea pacifica
Mixture
Beroe sp.
Ostracoda
Cyphocaris challengeri
Conchoecinae
Crustaceans
Thysanoessa sp.
Zoea
Eucalanus bungii
Copepoda
Calanus spp.
Neocalanus sp.
Taxon
0
2
4
18:3n3 (%)
Octopoda
Fish larvae
Chaetognaths
Tomopteris spp.
Beroe sp.
Paraeuchaeta elongata
Crangonidae
Clione limacina
Primno abysallis
Themisto pacifica
Limacina helicina
Euphausia pacifica
Thysanoessa sp.
Mixture
Megalopa
Munida quadrispina
Pleurobrachia sp.
Pasiphaea pacifica
Copepoda
Jelly mix
Scina borealis
Hyperia sp.
Ostracoda
Crustaceans
Pandalus spp.
Cyphocaris challengeri
Conchoecinae
Zoea
Calanus spp.
Neocalanus sp.
Eucalanus bungii
Taxon
0
10
20
30
DHA (%)
Thysanoessa sp.
Crangonidae
Beroe sp.
Megalopa
Euphausia pacifica
Limacina helicina
Clione limacina
Pandalus spp.
Munida quadrispina
Eucalanus bungii
Calanus spp.
Octopoda
Mixture
Chaetognaths
Themisto pacifica
Zoea
Primno abysallis
Copepoda
Jelly mix
Hyperia sp.
Pasiphaea pacifica
Crustaceans
Tomopteris spp.
Fish larvae
Scina borealis
Ostracoda
Paraeuchaeta elongata
Pleurobrachia sp.
Conchoecinae
Cyphocaris challengeri
Neocalanus sp.
Taxon
10
20
30
EPA (%)

Supplementary figure S4. $\delta^{13}C$ (A) and $\delta^{15}N$ (B) values of POM and zooplankton size classesin relation to latitude in the Strait of Georgia (SoG). Colours correspond to the three defined SoG regions.

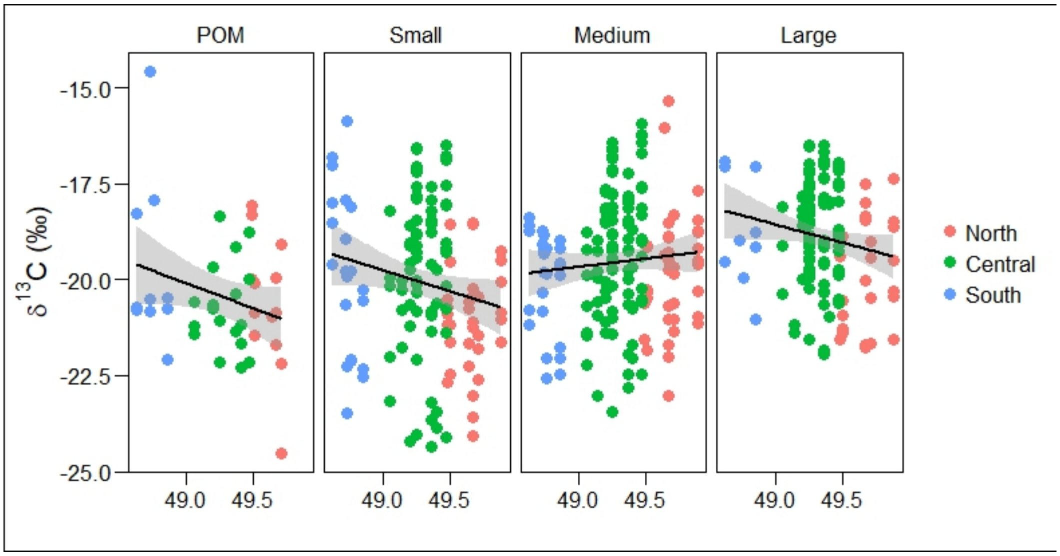

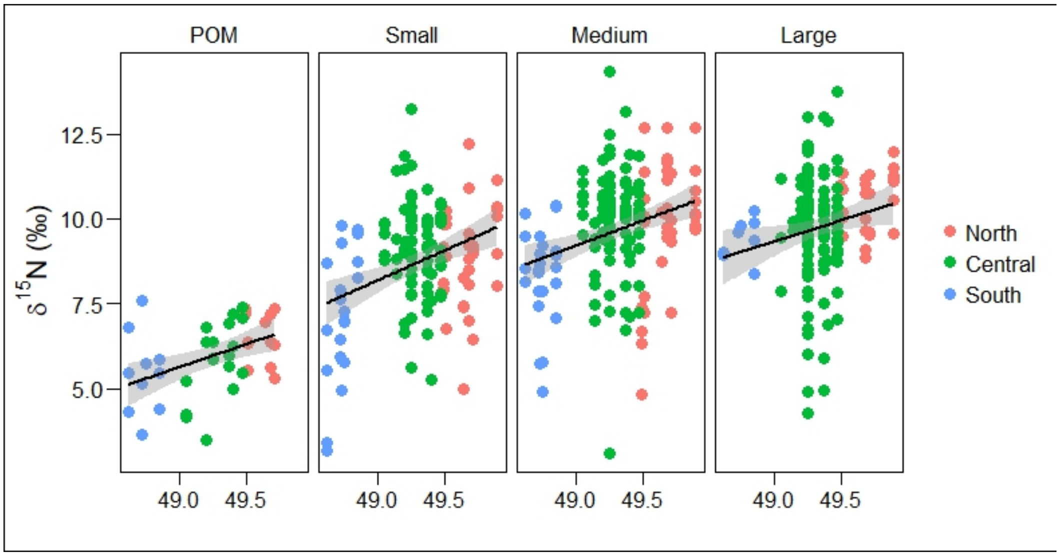

Supplementary figure S5. Biplots of C and N isotopic composition of POM and the analyzed zooplankton species in spring and summer. Grey dotted lines indicate trophic levels (TL) 1, 2 and 3 from bottom to top in each season.

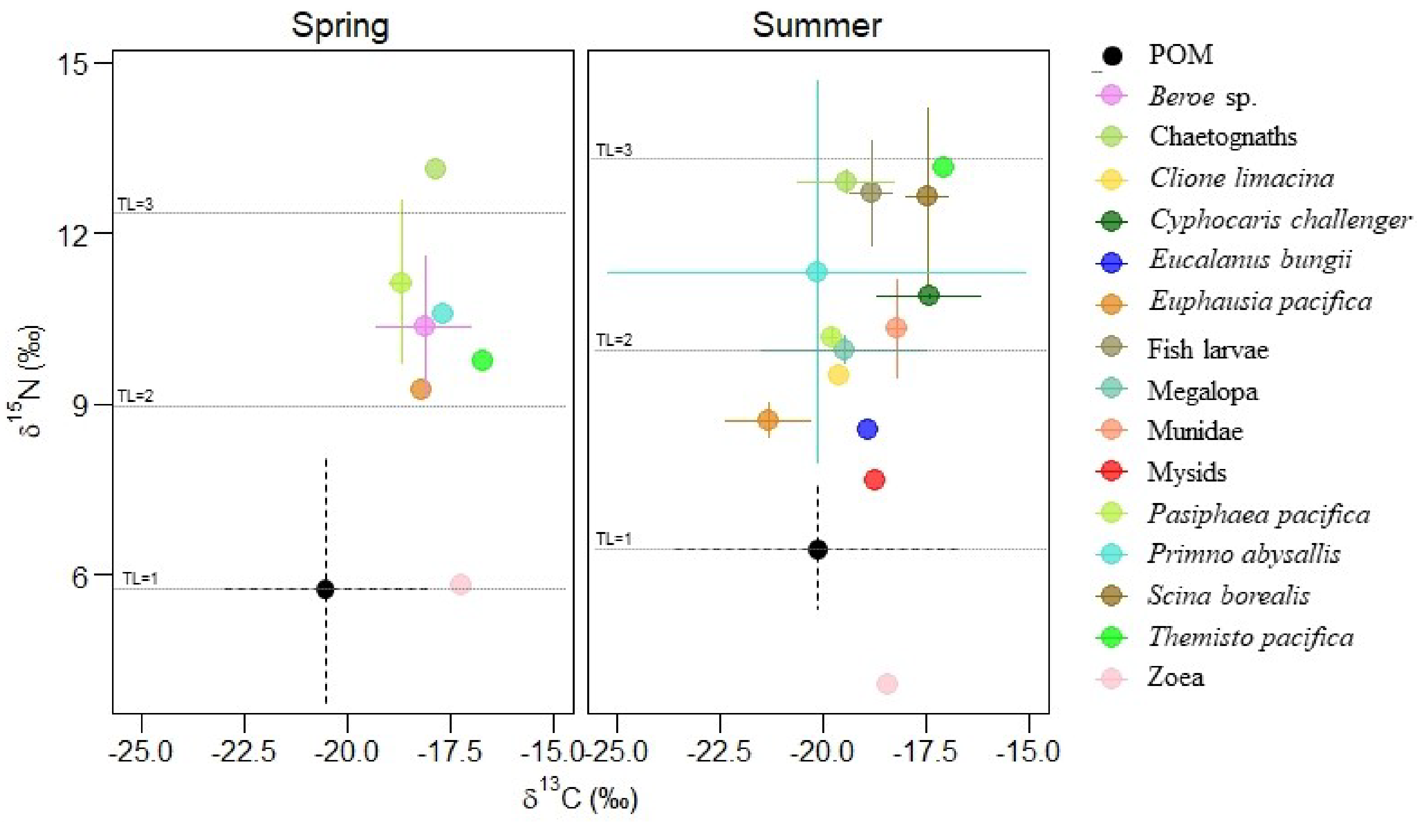

Supplement: Supplementary file 1 — Supplemental information. [file 41598_2020_65557_MOESM1_ESM.pdf]
